# Revisiting the importance of model fitting for model-based fMRI: It does matter in computational psychiatry

**DOI:** 10.1371/journal.pcbi.1008738

**Published:** 2021-02-09

**Authors:** Kentaro Katahira, Asako Toyama

**Affiliations:** Department of Psychological and Cognitive Sciences, Nagoya University, Nagoya, Japan; Seoul National University, KOREA, REPUBLIC OF

## Abstract

Computational modeling has been applied for data analysis in psychology, neuroscience, and psychiatry. One of its important uses is to infer the latent variables underlying behavior by which researchers can evaluate corresponding neural, physiological, or behavioral measures. This feature is especially crucial for computational psychiatry, in which altered computational processes underlying mental disorders are of interest. For instance, several studies employing model-based fMRI—a method for identifying brain regions correlated with latent variables—have shown that patients with mental disorders (e.g., depression) exhibit diminished neural responses to reward prediction errors (RPEs), which are the differences between experienced and predicted rewards. Such model-based analysis has the drawback that the parameter estimates and inference of latent variables are not necessarily correct—rather, they usually contain some errors. A previous study theoretically and empirically showed that the error in model-fitting does not necessarily cause a serious error in model-based fMRI. However, the study did not deal with certain situations relevant to psychiatry, such as group comparisons between patients and healthy controls. We developed a theoretical framework to explore such situations. We demonstrate that the parameter-misspecification can critically affect the results of group comparison. We demonstrate that even if the RPE response in patients is completely intact, a spurious difference to healthy controls is observable. Such a situation occurs when the ground-truth learning rate differs between groups but a common learning rate is used, as per previous studies. Furthermore, even if the parameters are appropriately fitted to individual participants, spurious group differences in RPE responses are observable when the model lacks a component that differs between groups. These results highlight the importance of appropriate model-fitting and the need for caution when interpreting the results of model-based fMRI.

## Author summary

Computational modeling has been utilized to infer the latent variables reflecting computational processes underlying behavior. This approach provides a predictor of neural activity by which one can test the physiological or neural measures that reflect computational processes. In this way, individual differences in physiological signals corresponding to latent variables (e.g., reward prediction error) and deficits related to mental disorders have been clarified. However, if the model contains a systematic error, the results may be erroneous. In this study, we suggest that spurious between-group (individual) differences are observable if a model parameter (e.g., learning rate) is misspecified. This bias may account for the inconsistencies in a series of studies that have addressed aberrant brain activity underpinning reward prediction error in patients with psychiatric disorders.

## Introduction

Computational modeling has contributed to the analysis of behavioral and physiological data in psychology, neuroscience, and psychiatry. One advantage of computational modeling is that it offers trial-by-trial estimates of latent variables underlying behavior [1]. The latent variable can be used as a regressor (predictor) for exploring corresponding physiological activity. One notable application is model-based functional magnetic resonance imaging (fMRI), wherein the brain regions that show correlated activity with latent variables are explored [2–6]. As a notable success of model-based fMRI, reward prediction errors (RPEs) have been associated with neural responses of the reward system involving dopamine such as the striatum [4, 7–9]. Other targets of application of model-based analysis include electroencephalogram (EEG) [10–12], electrophysiology [13, 14], and pupillometry [15]. Other than physiological activities, trial-by-trial behavioral measures (e.g., reaction time in subsequent trials) have been analyzed using such model-based regressors [16].

In model-based analysis, errors in parameter estimation and misspecification of model structure can be problematic. In general, the bias in parameter estimation and model misspecification can lead to erroneous conclusions [17–20]. However, Wilson & Niv [21] suggested that the error in model-fitting does not necessarily cause a serious error in model-based fMRI. Specifically, they developed a theoretical framework which enables quantification of how the misfit of the learning rate—an important parameter that determines the degree to which values are updated using RPE—affects the statistical significance of the regression coefficients for RPE or value. The authors reported that even in the most extreme cases, results do not substantially change, while this robustness can come at the expense of the ability to identify the specific function of a neural signal. However, Wilson & Niv’s theoretical framework did not deal with certain practical situations such as group comparisons, which we consider in the present study.

In the present study, extending the theoretical framework of Wilson & Niv [21], we report the situation where a slight error in parameter estimation has a substantial impact on model-based fMRI. Specially, we consider the situation focusing on individual (group) differences. For example, psychiatry studies have compared patients with a mental disorder (e.g., depression) and healthy controls. Deficits in representation of RPEs in patients have been reported [22–26]. However, the results have been inconsistent. Other studies reported comparable response to RPEs between healthy controls and patients with major depressive disorder [27, 28].

We demonstrate that even if RPE responses are completely intact in patients, a spurious difference to healthy controls can be observed. Such a situation occurs when the true learning rate differs between groups but a common learning rate is used to estimate RPE signals, as has been performed in previous studies [22, 23, 25, 29]. The use of a common parameter set is often employed in model-based fMRI to obtain stable regressors and thus robust estimates for neural activity [30]. While studies in computational psychiatry have focused on the difference in model parameters between subjects, the use of a common parameter set is still prevalent in model-based fMRI studies. The spurious effect due to parameter misestimation can also work in a dimensional approach, where researchers seek neural substrates whose activity is continuously correlated with, for example, the severity of psychiatric symptoms.

Furthermore, we demonstrate that even if the parameters are appropriately fitted to individual subjects, spurious group differences in RPE responses can appear when the model lacks a component that is contained in actual computational processes that differ between groups. The specific example we consider involves the forgetting process. The forgetting (decaying) of action values often significantly improves model-fitting [14, 31–33] but has not been included in previous studies that addressed diminished RPE in depression [22, 23, 28]. The forgetting rate was found to correlate with the tendency of depression [32]. Thus, unmodeled differences in the forgetting process may account for the apparent differences in neural responses to RPE.

Wilson & Niv [21] derived analytic expressions of regression coefficients and their statistics (t-value) using several approximations (See also S1 Text). However, their theoretical analysis lacks several practical factors. First, their analytical (mathematical) framework considered statistics of a single subject. Although statistical tests can be performed for single subjects in principle, group-level statistics (i.e., how a regression coefficient is distributed across subjects and how the distribution differs between groups) does matter in typical fMRI studies. Note that Wilson & Niv also showed group-level statistics as results for empirical fMRI data analysis, but they did not provide analytical treatment for those statistics. Second, Wilson & Niv’s analytical expressions considered general linear models (GLMs)—commonly used statistical models for fMRI—which contain only a single regressor, while GLMs used in model-based fMRI typically contain multiple regressors (e.g., reward magnitude, action value, and stimulus identity in addition to RPE). Indeed, two components of RPE, actual reward and expected reward, are often separately included in GLMs [34, 35]. The manner in which the parameter misfit affects results in such cases remains elusive. In the present study, we extend the theoretical framework by Wilson & Niv to address these issues.

## Results

### Illustration on classical conditioning task

We first illustrate how model-based fMRI works and how model parameters influence results based on the basic setting we consider in the present study. This section serves as an introduction of Wilson & Niv’s framework [21], which our theoretical considerations are based on. In addition, the simulation presented here helps us understand the mechanisms underlying the results, which we will report later on.

**Fig 1:**
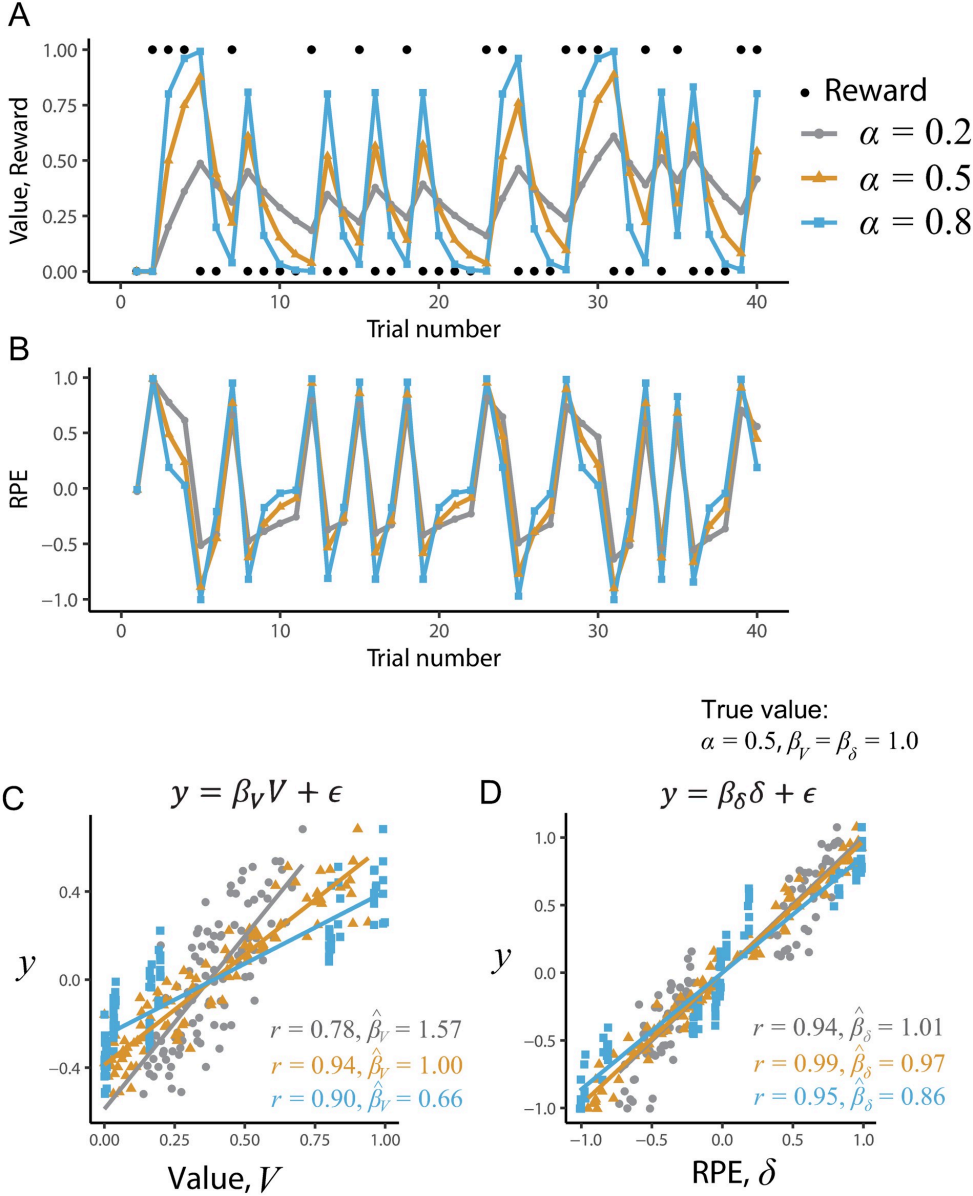
Examples of simulations of the Rescorla-Wagner model with varying learning rates, *α*. (A, B) Time courses of the value, *V*, and RPE, *δ*, respectively. (C, D) The effects of learning rate on the correlations between the value (C) / RPE (D) and hypothetical neural signal, which were generated by the linear regression model shown above each panel.

As an example model, we consider the Rescorla-Wagner (RW) model [36], which was also used by Wilson & Niv [21] as a basic reinforcement learning model. Here, we consider the situation where a subject passively experiences rewards associated with a neutral stimulus (i.e., classical conditioning paradigm; e.g., [2, 37]). At trial *t*, after a reward *r*_*t*_ is presented, a value *V*_*t*_ that represents the expectation of reward is updated according to:

$$V_{t+1}=V_{t}+\alpha \delta_{t},$$ (1)

$$\delta_{t}=r_{t}-V_{t},$$ (2)

where *δ*_*t*_ represents RPE and *α* denotes the learning rate, which determines the extent to which the RPE is reflected in the value of the next trial. The initial value of the value, *V*_1_, is set to 0, unless otherwise stated. In this model, the value, *V*_*t*_, and RPE, *δ*_*t*_, are latent variables that are determined given a reward sequence and the parameter, *α*, which may be fitted to data. Fig 1A and 1B show the simulated time courses of the latent variables for three different values of *α*. Note that here we chose excessively different learning rates (*α* = 0.2, 0.5, 0.8) to clarify the effect. In this simulation, the reward is delivered (*r*_*t*_ = 1) with the probability of 0.4, otherwise no-reward is given (*r*_*t*_ = 0). It is evident that the behavior of these latent variables depends on the learning rate, *α*: the greater *α* is, the greater the variances (magnitudes) of *V* and *δ*.

In model-based fMRI, estimates of latent variables such as value or RPE are used as a regressor (predictor) of BOLD signals (i.e., neural signals). Brain regions whose signals are significantly correlated with a latent variable are identified as regions that reflect the corresponding computational process. Specifically, this is performed by applying GLMs including the latent variable as regressors to the BOLD signals. For example, when the value signal, *V*, is of interest, the target signal at trial *t*, denoted by *y*_*t*_, is modeled using a GLM:

$$y_{t}=\beta_{V}V_{t}+\epsilon_{t},$$ (3)

where *ϵ*_*t*_ is noise variable. We assume that the noise obeys a Gaussian distribution whose mean is zero and variance is $\sigma_{\epsilon }^{2}$. *β*_*V*_ is the regression coefficient (the effect) of the value, *V*_*t*_. The estimate of the regression coefficient of value, is tested if it differs from zero (e.g., by one-sample t-test).

In the theoretical analysis of Wilson & Niv [21], the neural signal *y*_*t*_ is assumed to be generated by the GLM as per Eq 3 with the “ground-truth” regressor, *V*_*t*_, that is calculated based on a ground-truth model parameter (i.e., learning rate). Under this setting, Wilson & Niv attempted to evaluate the impact of mismatch between the ground-truth learning rate and the fit learning rate on the statistics of estimates for the regression coefficient (hereafter, we simply call the estimate as ‘beta value’). Specifically, they analytically calculated the correlation coefficient between the ground-truth regressor and that obtained with fit parameters (hereafter,‘fit regressor’). This correlation has a close relationship with the correlation between fit regressor and target neural signal: the stronger the correlation between fit regressor and ground-truth regressor, the stronger the correlation between fit regressor and neural signal. Thus, stronger correlations between true and fit regressors are related to larger regression coefficients of the fit regressor.

Fig 1C shows a correlation between a hypothetical neural signal, *y*_*t*_, and the value signal, *V*_*t*_. To generate *y*_*t*_, we assumed that the true learning rate is *α* = 0.5 and the true regression coefficient *β*_*V*_ = 1. When the fit learning rate, denoted by $\hat{\alpha }$, matches its true value (*α* = 0.5), the correlation coefficient between the value signal and hypothetical neural signal is larger (*r* = 0.94) than those with mismatched learning rates. However, even when the learning rate deviates largely from the true value, strong correlations are observed (*r* = 0.78 for $\hat{\alpha }=0.2$; *r* = 0.90 for $\hat{\alpha }=0.8$). In the case where $\hat{\alpha }$ matches its true value, the estimated regression coefficient, ${\hat{\beta }}_{V}$, is closest to the true value of 1 (${\hat{\beta }}_{V}=1.00$). Note that when the true learning rate is lower than the true value (*α* = 0.2, gray line), the beta value became even larger than the true value (${\hat{\beta }}_{V}=1.57$). This is because the variance of the value signal is smaller when the learning rate is small (compare the variances along x-axis in Fig 1C).

Fig 1D shows an example where the target signal reflects RPE:

$$\text{GLM1:}\;\;y_{t}=\beta_{\delta }\delta_{t}+\epsilon_{t},$$ (4)

where *β*_*δ*_ is the regression coefficient for RPE, and its true value is set to *β*_*δ*_ = 1. For the latter use, we refer to this model as GLM1. A strong correlation between the RPE signal and neural signal was observed (*r* = 0.99) when the fit learning rate matched its ground-truth value ($\hat{\alpha }=\alpha =0.5$). Compared to the case where the signal reflects the value (Fig 1C), the correlation coefficient and regression coefficient are less sensitive to a mismatch in learning rate: even when the fit learning rate $\hat{\alpha }$ differed from the true learning rate, the correlation coefficients between RPE and neural signal, *y*_*t*_, did not drastically decrease (≥ 0.94). This is in accordance with the conclusions of Wilson & Niv [21] that claim the results of model-based fMRI are not necessarily sensitive to a mismatch of fit parameters.

In the following section, we will demonstrate cases where a minute mismatch in parameter estimates leads to substantial errors, thus yielding erroneous conclusions.

### Group comparison on classical conditioning task

Here we consider a situation where neural responses to RPE (measured by beta values) during a classical conditioning task in two groups are compared. We concentrate on the RPE signal, which has been subjected to group comparisons (e.g., between patients and healthy controls) especially in psychiatry [22–24, 29, 38].

In the first scenario, we simulated an experiment in which reward is provided with a specific reward contingency in a classical conditioning paradigm as in the previous section. The trial-by-trial RPE was modeled by the RW model (Eqs 1 and 2). The neural signal, *y*_*t*_, was simulated using the GLM1 (Eq 4) where RPE was generated with the ground-truth learning rate that differs between groups. We consider a specific scenario where the neural responses to RPE are compared between subjects in a patient group whose learning rate is low (Low-L group; *N* = 20) and healthy controls (High-L group; *N* = 20) [22, 23, 29]. We assume the true learning rate of Low-L group (patient group) is *α* = 0.2, and that of High-L group (healthy control) is *α* = 0.4. This setting is in accordance with studies reporting that the learning rate is smaller in individuals with depression [39] (see [40, 41] for reviews). Crucially, the ground-truth value of the RPE regression coefficient did not differ between groups (*β*_*δ*_ = 1.0). This corresponds to the assumption that even in the patient group (Low-L group), the neural responses to RPE are completely intact. Only one behavioral characteristic, which is represented by learning rate, *α*, differed to that of healthy controls. As has been performed in [22–24, 29, 38], we assumed that a common parameter value (here we used $\hat{\alpha }=0.3$, i.e., the mean value of the true learning rates) was used for both groups to derive regressors for GLMs. We chose this value following the study that used the classical conditioning paradigm [29], where the common learning rate was set to $\hat{\alpha }=0.348$. However, as shown in S3 Text, the effect we report does not much depends on the value of each learning rate given the relative difference in learning rate between groups. The GLMs with the regressors are fit to the hypothetical neural data. Then, statistical analysis is performed to examine whether the beta values for the regressors differed between groups. We also report the effect size of a group difference in mean beta value (Cohen’s *d*), which is a key measure in our theoretical consideration. We attempt to evaluate the impact of the mismatch between ground-truth learning rate and fit learning rate on the effect size. Below, we will report the results with different GLMs being used as fit models.

#### GLM1

**Fig 2:**
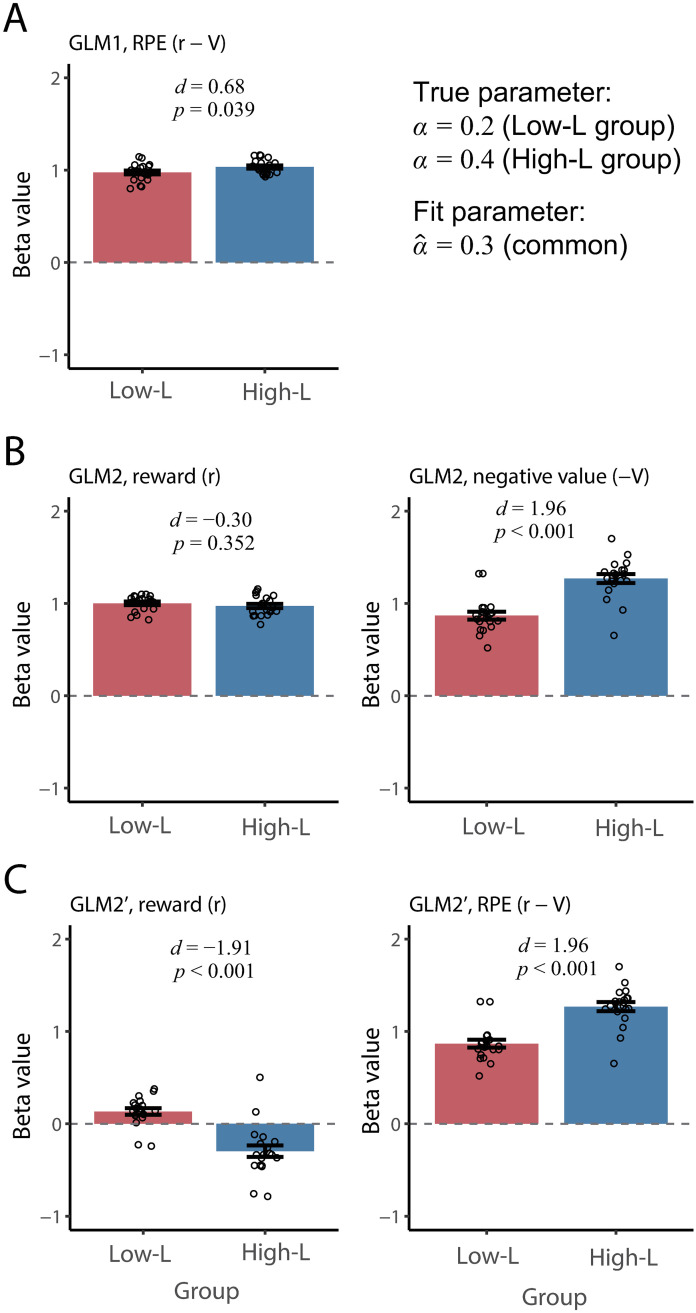
Simulations of group comparison of beta values (regression coefficients). GLMs are fit to the hypothetical data that are generated based on the RW model with different learning rates between groups. (A) GLM1 (RPE is a sole regressor). (B) GLM2 (reward and negative value are regressors). (B) GLM2′ (reward and RPE are regressors). The error bars indicate the standard error of mean (s.e.m). The result of unpaired t-test (p-value) and Cohen’s *d*, which quantify the effect size of group difference, are indicated in each panel.

First, we consider the result where GLM1 (Eq 4), in which RPE is the only regressor of interest, is applied to the simulated data. Fig 2A plots beta values (the estimates of the regression coefficient) for RPE, ${\hat{\beta }}_{\delta }$. Although the true regression coefficient was identical between both groups by construction (*β*_*δ*_ = 1.0), its estimate, ${\hat{\beta }}_{\delta }$, differed significantly between the two groups (*t*_38_ = 2.14, *p* = 0.039, unpaired t-test; Cohen’s *d* = 0.68).

#### GLM2

As RPE (*δ* = *r* − *V*) is correlated with the reward signal (*r*) itself, a non-zero regression coefficient (positive correlation) for RPE may indicate that the target signal only includes the effect of the reward itself, independently of reward prediction. Several studies have addressed this issue by separately including two components of RPE, that is, reward *r* and negative value, −*V*, as regressors into the GLM [34, 35]. Here, we refer to such a model as GLM2:

$$\begin{array}{c} \text{GLM2:}\;\;y_{t}=\beta_{r}r_{t}+\beta_{NV}\left(-V_{t}\right)+\epsilon_{t}. \end{array}$$ (5)

To claim that the target signal reflects RPE, significantly positive beta values for both regressors (*β*_*r*_ and *β*_*NV*_) are required [34, 35]. The true model in the simulation corresponds to the GLM2 with *β*_*r*_ = 1 and *β*_*NV*_ = 1.

Fig 2B shows beta values, ${\hat{\beta }}_{r}$ and ${\hat{\beta }}_{NV}$ in GLM2 for the same synthesized data as those in GLM1. A significant group difference in the beta values was observed for the negative value (${\hat{\beta }}_{NV}$; *t*_38_ = 6.19, *p* < 0.001, Cohen’s *d* = 1.96) but not for the reward signal (${\hat{\beta }}_{r}$; *t*_38_ = −0.94, *p* = 0.352, Cohen’s *d* = −0.30). The group difference in ${\hat{\beta }}_{NV}$ was clearly larger than that of beta value for RPE in GLM1 (Fig 2A). This is because the beta value for RPE represents the mixed effect of the reward signal, which does not differ between groups, and the negative value, which is subject to the effects of parameter misfitting. In GLM2, the latter effect is isolated as ${\hat{\beta }}_{NV}$. This effect of isolation is also manifested in the difference in correlation coefficients between Fig 1C (between *V* and *y*) and Fig 1D (betwen *δ* and *y*).

While the present study focuses on the RPE signal, a number of model-based fMRI studies have focused on the (expected) value signal (e.g., [9, 23]). Such case of value representation can be modeled by using Eq 3 as a model of neural activity. Our results basically apply to this case, because we set the reward signal (*r*) as known regressor (and thus set to the true value), the beta value for the negative value signal (*β*_*NV*_) in GLM2 has a similar estimate for the value signal (*β*_*V*_) in case of value representation, given that the correlation between reward and negative value is negligible.

#### GLM2′

Several studies have used a GLM that incorporates both the reward signal and RPE as separate regressors [9, 42–45]. In the present study, we represent such a model as GLM2′:

$$\begin{array}{c} {\text{GLM2}}^{\prime }:\;\;y_{t}=\beta_{r^{\prime }}r_{t}+\beta_{\delta^{\prime }}\delta_{t}+\epsilon_{t}. \end{array}$$ (6)

The true model in the simulation can be represented with *β*_*r*′_ = 0 and *β*_*δ*′_ = 1 in GLM′.

Fig 2C shows the beta values in GLM2′. Note that ${\hat{\beta }}_{\delta^{\prime }}$ in GLM2′ has almost the same value as ${\hat{\beta }}_{NV}$ in GLM2 (Fig 2B). This can be explained as follows. GLM2′ (Eq 6) can be rewritten as *y*_*t*_ = *β*_*r*′_ *r*_*t*_ + *β*_*δ*′_ (*r*_*t*_ − *V*_*t*_) + *ϵ*_*t*_, which leads to *y*_*t*_ = (*β*_*r*′_ + *β*_*δ*′_)*r*_*t*_ + *β*_*δ*′_(−*V*_*t*_) + *ϵ*_*t*_. This can be equivalent to GLM2 with the relation that (*β*_*r*′_+ *β*_*δ*′_) in GLM2′ corresponds to *β*_*r*_ in GLM2 and *β*_*δ*′_ in GLM2′ corresponds to *β*_*NV*_ in GLM2.

GLM2′ exhibits the issue of strong multicollinearity, often discussed in the statistics literature—whereby strong correlations among regressors (here, between reward and RPE) render the estimates unreliable [46]. Indeed, the variance of the beta value of reward, *β*_*r*′_, of GLM2′ (Fig 2C) was larger than *β*_*r*_ of GLM2 (Fig 2B). Furthermore, in GLM′, the effect of the reward is represented by the sum of two regression coefficients (*β*_*r*′_ + *β*_*δ*′_), rather than a single regression coefficient for reward. Thus, the interpretation of the regression coefficient is not straightforward. Compared to GLM2, GLM2′ does not provide further information. Thus, GLM2 is preferable and we do not further consider GLM2′ in the present study.

#### Theoretical analysis

Based on Wilson & Niv [21], we conducted a theoretical analysis to attain insight into the mechanisms underlying the above results. Specifically, we analytically calculated the statistics of the beta values of the GLMs, which enabled us to evaluate the effect size of (spurious) group differences. Based on the analytically obtained effect size, we can also evaluate how frequently (spurious) statistically significant differences between groups are obtained (i.e., statistical power, which is the probability that a statistically significant result would be obtained; see Materials and methods for details).

In the case of comparisons between means of regression coefficients of both groups, the effect size can be measured by

$$\begin{array}{c} d_{2}=\frac{\text{E}\left[{\hat{\beta }}^{\left(1\right)}\right]-\text{E}\left[{\hat{\beta }}^{\left(2\right)}\right]}{\sqrt{(\text{Var}\left[{\hat{\beta }}^{\left(1\right)}\right]+\text{Var}\left[{\hat{\beta }}^{\left(2\right)}\right])/2}}, \end{array}$$ (7)

where E[⋅] indicates the expected value, Var[⋅] indicates the variance, and ${\hat{\beta }}^{\left(1\right)}$ and ${\hat{\beta }}^{\left(2\right)}$ indicate the beta values of interest for the first group and the second group, respectively. In the present study, we assume that the healthy-control group (High-L group and Low-F group in the later scenario with model-misspecification) corresponds to the first group and the patient group (Low-L group and High-F group) corresponds to the second group so that the positive *d* indicates diminishment of beta value in the patient group compared to the control group. This effect size measures difference in the population means between groups with the unit of their standard deviation. Cohen’s *d* is one of the estimators for this effect size. Given a sample size (the number of subjects for each group, *N*), the effect size, *d*_2_, determines the statistical power; larger the magnitude of *d*_2_ is, the larger the power (see Materials and methods).

**Fig 3:**
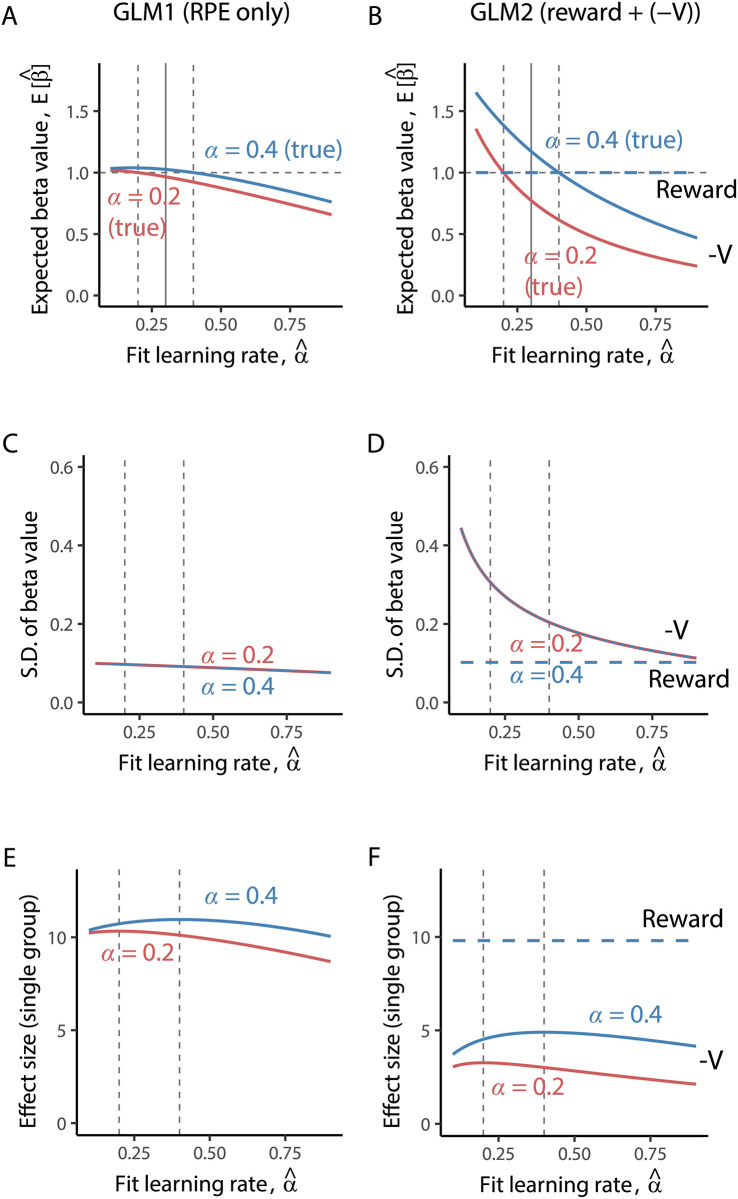
Influence of misfit of model parameters on beta values for the Rescorla-Wagner model. (A, B) The expected value of beta values in GLM 1 (A) and GLM2 (B), obtained using Eqs 39, 41 and 43. The fit learning rate used for the simulation in Fig 2, $\hat{\alpha }=0.4$, is indicated by the solid vertical lines. (C, D): The standard deviation of beta values in GLM 1 (C) and GLM2 (D). These are obtained by taking the square root of Eqs 40, 42 and 44. (E, F): The effect size (*d*_1_; for single group) of beta values in GLM 1 (E) and GLM2 (F). In all panels, the red lines represent the results for the true learning rate *α* = 0.2 and blue lines represent *α* = 0.4. In panel A, C, and E, these lines represent the results of the regression coefficient of RPE, *β*_*δ*_. In panel B, D, and F, they represent the results of the regression coefficient for negative values, *β*_*NV*_. Broken bold lines indicate the results of regression coefficient for reward, *β*_*r*_.

The expected value and variance (or s.d.) of beta values for both groups are required to calculate the effect size, *d*_2_ (Eq 7). The analytical expression of these quantities are given as Eqs 39–48 in Materials and methods. Specific values of these statistics are plotted in Fig 3 as a function of the fit learning rate, $\hat{\alpha }$. For the case in Fig 2 ($\hat{\alpha }=0.3$), the effect size for RPE in GLM1 is *d*_2_ = 0.64, which yields the statistical power of 50.2% with a significance level of 0.05 and sample size of *N* = 20 (for each group).

The effect size of the group differences in ${\hat{\beta }}_{r}$ in GLM2 is *d*_2_ = 0 which leads to a statistical power of 5%, the pre-defined type-I error rate (the significance level). This is because the expected value of the beta value for reward is not influenced by the RL model parameter and is identical for both groups. On the other hand, the statistical power for detecting differences in *β*_*NV*_ in GLM2 is 99.9% with *d*_2_ = 1.64. Thus, the spurious difference in response to a negative value, −*V*, is easily observed with this setting.

The reason that the beta value is larger for a larger true learning rate (compare red line for *α* = 0.2 and blue line for *α* = 0.4, Fig 3A and 3B) is explained as follows. As can be observed in our simulation (Fig 1B), a larger learning rate leads to larger variance of RPE. Thus, using a smaller learning rate compared to the true value leads to smaller variance of the regressor. It should be noted that this depends on the reward contingency. In cases where reward mean drifts and the variance of reward around the reward mean is relatively small, the opposite can occur; using a larger learning rate compared to the true one leads to a smaller variance of RPE since the model with a smaller learning rate cannot follow the changing reward rate. See S4 Text for specific examples. To compensate for the smaller variance of the fitted RPE, the corresponding beta value has a larger value. If the true value of the learning rate is used, the expected value of beta value has no bias (equals the true regression coefficient, 1; Fig 3A and 3B). This suggests that using a more accurate parameter for both groups (and for all subjects) can reduce the risk of a spurious group difference.

To examine whether the target BOLD signal reflects the signal of interest (e.g., RPE signal), a one-sample t-test that examines whether the regression coefficient differs from zero is performed in second-level (or group-level) analysis of fMRI data (see Materials and methods). Such analyses are often independently performed for each group in psychiatric research. In these studies, significant activity (non-zero regression coefficient) in the control group and its absence in the patient group have been reported [22–24]. The effect size for this one-sample t-test is measured by the expected value of the beta value (Fig 3A and 3B) over its standard deviation (Fig 3C and 3D):

$$d_{1}=\frac{\text{E}[\hat{\beta }]}{\sqrt{\text{Var}[\hat{\beta }]}}.$$ (8)

Analytical evaluations of this effect size are shown in Fig 3E and 3F. The effect sizes for *β*_*δ*_ and *β*_*NV*_ adopt the maximum value when the fit learning rate matches the true parameter (indicated by the vertical dashed lines). Crucially, for any values of the fit learning rate, the effect size for a higher true learning rate (*α* = 0.4) is always larger than that for a lower true learning rate (*α* = 0.2). This suggests that the regression coefficient for RPE is easily deemed significant for the group with a higher true learning rate compared to those with a lower true learning rate, irrespective of the fit learning rate. This property may result in neural activity in a patient group (with a small learning rate) showing no significant response to RPE (or negative value), while the healthy control group shows a significant response.

### Factors that influence effect size of group difference

Next, we investigated the several factors that influence the between-group effect size, including the number of trials, within-group heterogeneity, and reward contingency.

#### Effect of number of trials

Our analytical result revealed that the effect size of the (spurious) group difference depends on the number of trails, *T*, in addition to the true learning rate and fit learning rate (Eqs 40 and 42). Here we examine the effect of the number of trials on the between-group effect size. We also considered the effect of a difference in true learning rates between groups (*α*_2_ − *α*_1_). In S3 Text, we report the results of the systematic simulation where we examined all possible combinations of learning rate. We found that the effect size mainly depends on the relative group difference in learning rates, rather than their absolute values. Given this result, here we focus on the difference in the learning rate while we fixed the mean (fit) learning rate to 0.3.

**Fig 4:**
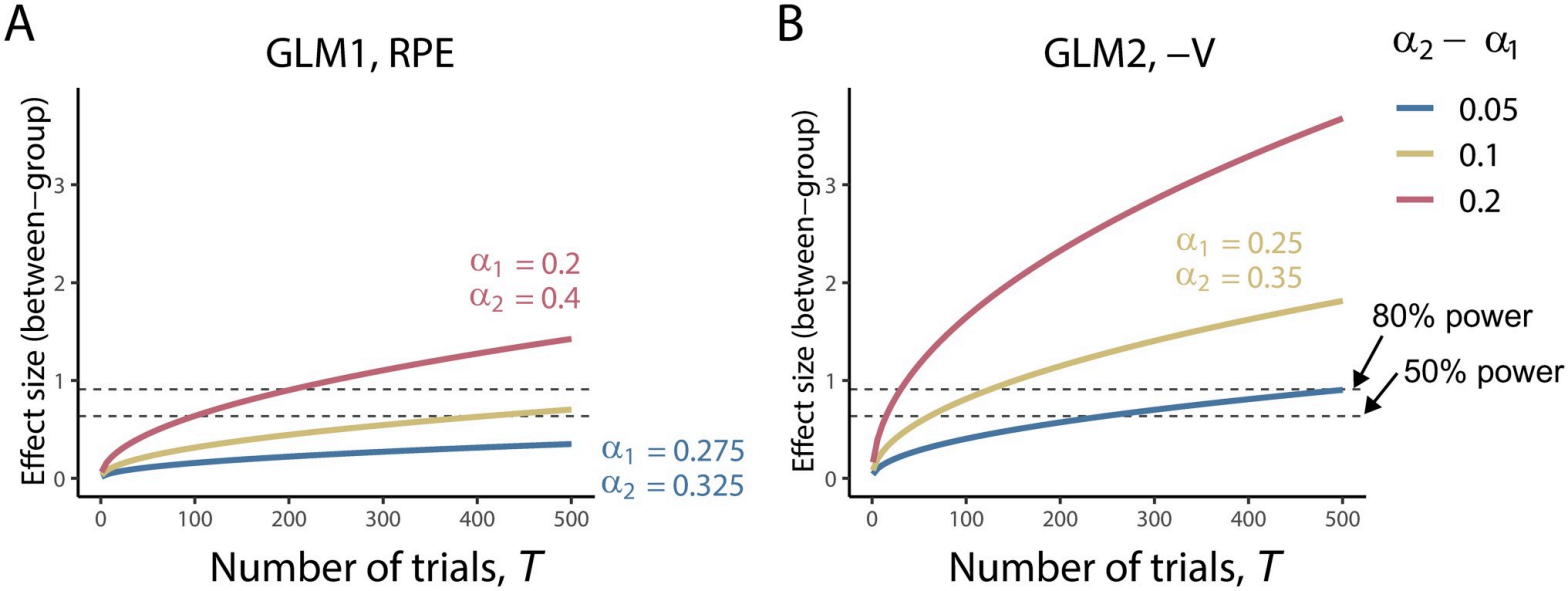
Dependence of between-group (spurious) effect size on the number of trials and between group differences in learning rates. (A) For beta values of RPE, *δ*, in GLM. (B) For beta value of negative value, −*V*, in GLM2. The fit learning rate is set to $\hat{\alpha }=0.3$ for all cases. For reference, we plot horizontal dot lines indicating the effect sizes above which statistical power is higher than 50% and higher than 80% with a significance level of 0.05 and sample size of *N* = 20 (for each group).

In Fig 4, we plot the between-group effect size as a function of the number of trials for various combinations of true learning rates. The results show that, as the number of trials increases, so does the effect size. Notably, the increase rate is larger when *T* is relatively small (e.g., *T* < 100); for a larger *T*, the increase is rather moderate. As the difference in learning rate increases, the between-group effect size also increases.

It should be noted that the analytic expressions obtained following Wilson & Niv [21] rely on an approximation that holds for a large number of trials (due to the ignorance of initial transient phase). Thus, we should be careful when applying this expression to cases with a small number of trials. To check the analytical expressions of the effect size in the range of *T* considered here, we compared the results of simulations and analytical expressions. Simulations were performed 100 times for each number of trials for cases with *α*_1_ = 0.2 and *α*_2_ = 0.4. The results are shown in S1 Fig. The analytical results (blue lines) well agree with those obtained from the simulations (symbols), which validates the analytical calculations.

#### Effect of within-group heterogeneity

We have supposed learning rates are identical (homogenous) within each group. Although this assumption of homogeneity is unrealistic, we adopted it to simplify the interpretation and analytical calculation. To examine the effects of within-group differences on group-comparisons of beta values, we performed simulations where the within-group variance of true learning rates was systematically varied and the effect size of the group difference numerically calculated.

**Fig 5:**
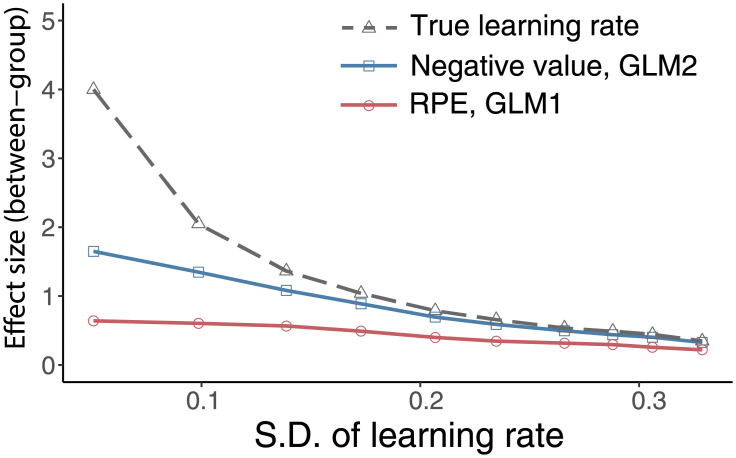
The effects of within-group differences in true learning rates on beta values (regression coefficients) and true learning rate. The between-group effect size of learning rate (gray), RPE of GLM1 (red), and negative value of GLM2 (blue) are plotted as a function of the standard deviation (s.d.) of true learning rate. The s.d. of learning rate is calculated for each group and then averaged across groups. Note that the s.d. is calculated for the actual parameter value after the truncation that restricts the range of learning rate to [0, 1].

Fig 5 shows the results. The effect size of group differences in true learning rates were also computed (gray line). As the within-group s.d. of true learning rates increases, the effect sizes of group differences in beta values for RPE (red line) and negative value (blue line) decrease. However, these decreases are rather moderate. Thus, the assumption of homogeneity does not influence qualitative results, which we report in the present study.

As the s.d. of true learning rates increases, the effect size of learning rates approximates the effect size of beta values. It should be noted that when the estimates of learning rate contain an estimation error, its effect size becomes smaller than shown in this simulation. This implies that even when no significant group difference in learning rate is detected, spurious differences in beta value for RPE can be observed. This suggests that the absence of a significant difference in learning rates between groups is insufficient to validate the use of common learning rates for both groups.

#### Effect of reward contingency

So far, we have considered the situation where the reward contingency is stable (i.e., the reward probability was fixed to 0.4). While such a fixed reward contingency has been often employed in model-based fMRI studies [2, 8, 35], different reward contingencies have also been often used in the experimental design for model-based fMRI [9, 34]. Wilson & Niv [21] considered the effects of reward contingencies on beta values in their analytical calculations. Their analysis suggests that the autocorrelation of reward sequences (which is zero in our fixed reward probability) does matter: autocorrelation makes the RPE signal or value signal insensitive to the misfit of learning rate in certain situations.

In the S4 Text, we explored the effect of reward schedule on the effect sizes (Cohen’s *d*) regarding the spurious differences between beta values for RPE and negative value. Specifically, we examined typical reward contingencies, those with drifting reward mean with Gaussian (continuous) reward, drifting reward probabilities with binary reward, and switching reward probability. Overall, the results indicated that as the autocorrelation of reward sequence increases, the effect sizes decreased or even reverted (i.e., Low-L group, where the true learning rate is smaller than High-L group, showed the larger beta values). See S4 Text for a detailed explanation on this issue.

### Reward sensitivity

Several studies reported that the subjective reward magnitude is smaller in individuals with depression [47, 48]. Such effect is represented in reinforcement learning models by introducing a reward sensitivity parameter [47]. With the reward sensitivity parameter *ρ*, the RPE in the RW model (Eq 2) becomes:

$$\delta_{t}=\rho r_{t}-V_{t}.$$ (9)

Note that ‘temperature parameter’ or decision stochasticity parameter has the equivalent role as *ρ* in guiding choice behavior.

In S2 Text, we examined the effect of reward sensitivity, *ρ*, on the effect size of group comparisons. Similarly to the effect of learning rates, the use of the common estimate $\hat{\rho }$ for two groups when true *ρ* differs between them leads to a biased estimate of the regression coefficient for RPE, *β*_*δ*_. Crucially, the true reward sensitivity *ρ* can substitute exactly for the true regression coefficient, *β*_*δ*_, as can be confirmed in Eq 39, i.e., doubling the true *ρ* has an identical effect to doubling the true regression coefficient on the predicted neural activity. These are in principle not distinguisable from data. Such differences between groups may be better interpreted as true differences in RPE effects on neural activity, rather than spurious differences due to parameter misfit. In contrast, when the different estimates between groups are used as reward sensitivity, $\hat{\rho }$, the beta values (${\hat{\beta }}_{r}$, ${\hat{\beta }}_{\delta }$, and ${\hat{\beta }}_{NV}$) show between-group differences even if the true *ρ* is identical between groups. As such, it is not recommended to use reward sensitivity parameters for group comparison in model-based fMRI. Indeed, most RL models used for model-based fMRI include the inverse temperature parameter instead of reward sensitivity (Eq 50 in Materials and methods). The inverse temperature parameter has identical effects with reward sensitivity on choice behavior [47, 49], but has no effect on the magnitude of the RPE signal, unlike reward sensitivity.

### Dimensional approach

Thus far, we have considered the situation of group comparisons. The field of mental health and computational psychiatry is increasingly focusing on dimensional approaches [50, 51], i.e., researchers seek continuous moderators for psychiatric symptoms. Dimensional approaches are known to be able to attain high statistical power compared to categorical approaches [52–54].

**Fig 6:**
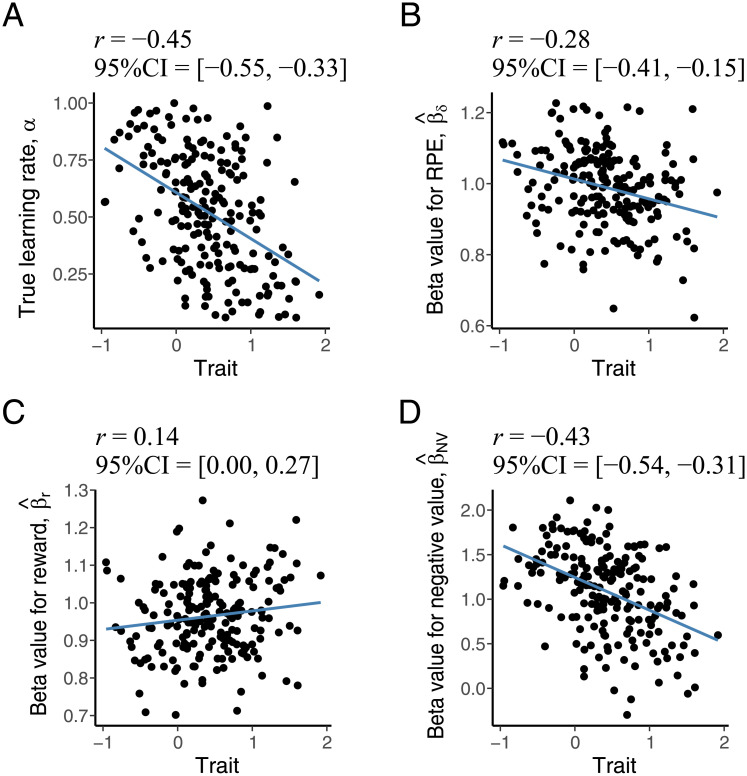
Spurious individual difference on model-based fMRI can occur in dimensional approaches (focusing on continuous trait, rather than group difference). (A) Simulated data showing correlation between trait (e.g., symptom score) and ground truth learning rate. (B) Correlation between trait and beta values for RPE in GLM1. (C, D) Correlation between trait and beta values for reward (C) and negative value (D) in GLM2. Above each panel, *r* indicates the correlation coefficient and ‘’95% CI” indicates the 95% confidence interval of the correlation coefficient.

By considering a simple scenario, we illustrate how the spurious effect of the model misfit can also work in dimensional analysis for model-based fMRI (e.g., [38]). In this simulation, we assume that the ground-truth learning rate in the RW model negatively correlates with some trait (e.g., a symptom score of depression; Fig 6A) across 200 subjects (here we assumed a relatively large number of subjects to obtain stable estimates for correlation coefficients between the beta values and the trait). The common learning rate ($\hat{\alpha }=0.4$) is supposed to be used for all subjects. Other conditions are identical to the group comparison case in the classical conditioning task with a fixed reward probability.

The resulting correlation between the beta values and the trait are plotted in Fig 6B–6D. The true regression coefficients for RPE were set to 1, as in the group comparison case. This means that the neural activity reflecting RPE has no relation to the trait of interest. However, a significant negative correlation between the beta value for RPE (in GLM1) and the trait was observed. In GLM2, the beta value for the negative value also showed a significant negative correlation with the trait. This demonstration highlights that the spurious effect on the model-based fMRI caused by the ignorance of individual differences in behavior can also occur in dimensional approaches.

### Effect of model-misspecification

Thus far, we have discussed the effects of parameter misspecification where the model structure is assumed to be true. In reality, there may be cases where the model structure does not correctly capture the true underlying processes, an issue called model-misspecification [17–20]. Here, we consider the effect of model-misspecification on the estimates of regression coefficients in GLMs. Previous studies have revealed that choice behavior of humans and other animals is better explained by a model with a forgetting component [14, 31]. The forgetting component is modeled by updating the action value for unchosen option as follows:

$$V_{t+1}=V_{t}+\phi \left(\mu -V_{t}\right),$$ (10)

where *ϕ* is the forgetting rate and *μ* is the default value [32, 33]. *μ* is set zero in most previous models [14, 31]. A recent study showed that this forgetting component may have a potential to characterize certain psychiatric characteristics [32]. Specifically, the study [32] reported that individuals with higher depressive symptom scores tended to show higher effects of a forgetting component which is described as a forgetting rate parameter in learning models. In addition, a recent theoretical study suggested that including this process can better explain dopamine responses related to value-learning and motivation [55].

By simulation, we synthesized datasets consisting of two groups with different forgetting rates: High-F group (*N* = 30; assuming depression group) with the forgetting rate being *ϕ* = 0.4 and Low-F group (*N* = 30; assuming healthy controls) with *ϕ* = 0.05. We compared cases for which the dataset was fit by a standard RL model without the forgetting component (misspecified model) and cases for which the data were fit by an RL model with this component (correctly-specified model).

**Fig 7:**
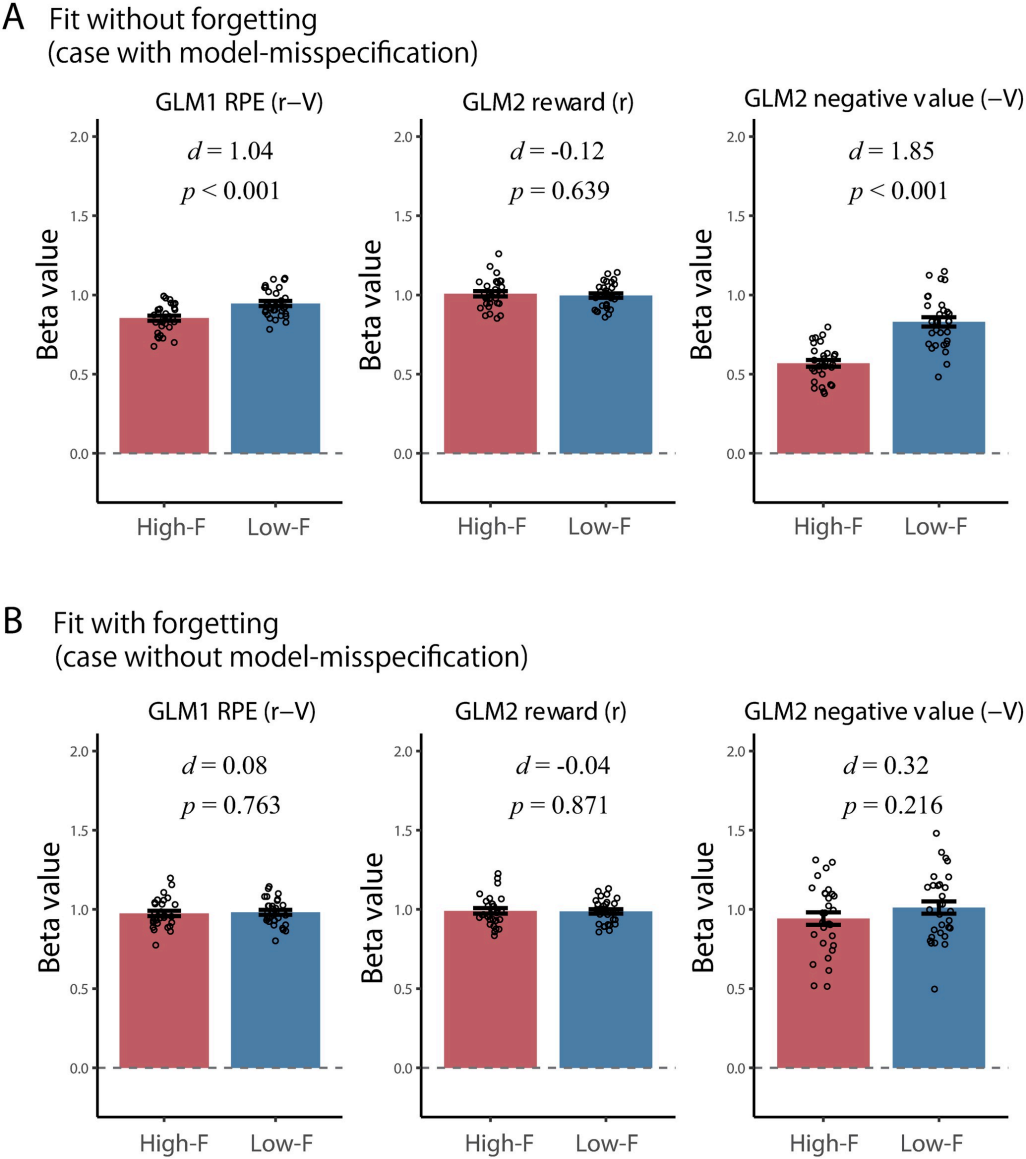
Effect of model-misspecification (lack of forgetting component). (A,B) Group comparisons of regression coefficients when the RL model without the forgetting component is used (A) and when the RL model with the forgetting component is used (B). In each panel, the beta values of GLM1 (RPE is a sole regressor) and GLM2 (reward and negative value are regressors) are shown. The error bars indicate s.e.m. The result of unpaired t-test (p-value) and Cohen’s *d* are indicated in each panel. High-F, the group with high forgetting rate (*ϕ* = 0.4), Low-F, the group with low forgetting rate (*ϕ* = 0.05).

Fig 7 shows the estimated beta values for the two groups using the RL model without the forgetting component, while the true model has this component (a case with model-misspecification). From left to right, each panel shows beta values of RPE in GLM1, reward in GLM2, and negative value in GLM2. For GLM1, although the (true) regression coefficients of RPE, *β*_*δ*_, were common to both groups, the beta values of the High-F group were assessed as significantly lower than those of Low-F group (*t*_58_ = 4.01, *p* < 0.001, unpaired t-test; Cohen’s *d* = 1.04). This is due to stronger model misspecification in the High-F group compared to that in the Low-F group, leading to decreased correlations between calculated RPE and hypothetical neural signals (*r* = .619 for High-F group and *r* = 0.675 for Low-F group, on average). For GLM2, the beta value of reward was not influenced by the lack of forgetting components (*t*_58_ = −0.47, *p* = 0.64, unpaired t-test; Cohen’s *d* = −0.12), while that of negative value was diminished in the High-F group compared to that in the Low-F group (*t*_58_ = 7.16, *p* < 0.001, unpaired t-test; Cohen’s *d* = 1.85) as the result of larger model misspecification. This result implies that, when a model-based fMRI study uses a standard RL model without a forgetting component as is often the case, it can easily report spurious differences due to model misspecification. In this simulation, we used a probabilistic reversal learning task in which the reversal of reward contingency between options occurred once at the middle of the experiment (see Materials and methods). In S2 Fig, we also examined the effect of the number reversals. Because the reversal emphasizes the effect of forgetting [31], as the number of reversals increased, the effect size of the between-group difference increased.

If the value and RPEs are calculated based on the RL model with the forgetting component, the group differences in beta values disappear (Fig 7B: *ps* > 0.22) with similar correlations between calculated RPEs and hypothetical neural signals (*r* = 0.667 for High-F and *r* = 0.680 for Low-F groups, on average). This indicates that including an appropriate component into the RL model reduces the risk of observing a spurious difference.

## Discussion

Model-based analyses using latent variables obtained from computational models (e.g., reinforcement learning models) are becoming an indispensable tool in neuroscience, psychology, and computational psychiatry. In such model-based analyses, the estimates of regression coefficients (beta values) for latent variables are often used as a proxy of physiological (e.g., BOLD signal) or behavioral responses that reflect latent variables. The quality of the estimates of regression coefficients depends on the parameter estimates of computational models (e.g., learning rate in reinforcement learning). Thus, an estimation error in the upstream level (i.e., in computational model-fitting) can influence downstream-level estimates (i.e., regression coefficient). In certain situations, the impact of the error may not be serious [21]. However, the present study showed cases where the effect leads to serious misleading consequences: when the regression coefficients were compared across subjects, the effect of estimation errors can yield spurious group or individual differences. Below, we discuss the implications of our results and provide recommendations for researchers to conduct a model-based analysis of fMRI or behavioral data.

The problem reported in the present study occurs when there is a systematic difference between parameter estimates and ground-truth parameter values (i.e., estimation bias), and the direction of the bias is associated with some trait of interest (e.g., severity of psychiatric symptoms). This situation likely occurs when common parameter values are used for all subjects, as commonly done in model-based fMRI to make the estimates stable [4, 5, 7, 22–25, 38, 43]. While this may help obtain stable regressors [30], our results indicate that this approach has an adverse effect: it causes systematic bias such that the regression coefficients for subjects with a higher (ground-truth) learning rate are under-estimated, while those for subjects with a lower learning rate are over-estimated. Note that this is the case for the fixed-probability reward case. This effect depends on reward contingency, as we discussed in S4 Text. This bias leads to spurious individual (between-group) differences when the neural response is compared between groups or subjects. The spurious difference can also appear when there is a certain level of within-group heterogeneity, although the heterogeneity reduces the effect. This spurious effect also occurs in a dimensional approach, where regression coefficients for each subject are correlated with the subject’s traits (e.g., the severity of psychiatric symptoms).

To avoid this bias, model parameters should be optimized in a less biased manner for individual subjects. If individual estimates for a single subject are unreliable (e.g., due to small trial number), the hierarchical modeling technique, which utilizes both population information and individual information, may be helpful [56–59]. However, hierarchical approaches can also be subject to statistical bias. For example, a hierarchical approach pulls individual parameter estimates towards the group mean, a property known as shrinkage. If one sets a common group-level distribution across the entire population (i.e., all subjects are assumed to be drawn from a common population), individual estimates are biased towards the mean of the entire population. Thus, the effect that causes spurious group differences, which the present study considered, can work albeit weakly compared to using a common fixed parameter. In contrast, if different group distributions are separately used for different groups, the effect of spurious group differences can be avoided [60, 61], but this might lead to spurious group differences in parameter estimates of the computational model [62, 63]. Another simple approach to avoid the potential problem reported here is to split subjects into two groups and fit a separate single parameter set to each group [64], which can be regarded as an extreme case of the separate-group hierarchical model. Appropriate model-fitting depends on the goal and would be an issue requiring future research.

We recommend that researchers report the results obtained with various levels of model-fitting approaches. For example, suppose one finds the group difference in beta values with common parameter estimates for entire subjects. In that case, checking the result remains with individually-fit model parameters would confirm that the result is not solely attributed to the parameter estimation bias. However, individual estimates for single subjects are often noisy, which might obscure the true effects. Thus, even if the group difference in the fMRI, observed in the whole-group analysis, disappears, the lack of the group-difference might not be attributed solely to the parameter estimation bias. As we discussed above, the hierarchical estimation may help reduce the estimation noise and recover the group difference in fMRI. Note that the hierarchical model may also induce a bias on model parameter estimates at the individual level, which depends on the group-level distribution as we discussed above. Thus, performing various hierarchical-models and checking how fMRI results are affected would be recommended.

Group comparisons of neural responses to RPE have been commonly explored in psychiatry research. As mentioned in the Introduction, blunted neural responses to RPE signal in individuals with depression have been addressed [22, 23]. However, inconsistent results have been reported: recent studies reported that comparable RPE responses with healthy controls were observed in patients with major depressive disorder [27, 28]. The results in the present study offer an account for this inconsistency. If the true learning rate differs between depressive individuals and healthy controls, the use of common parameters to both groups [22, 23] may cause a bias in regression coefficients of the RPE signal and lead to a spurious difference in the NAc response to RPE. On the other hand, in Rutledge et al. [27], a reinforcement learning model was not used, but the expected value of each option is was explicitly presented to the subjects; thus, the results may be free from the bias.

Even if model parameters are carefully fit to individual subjects, model misspecification can cause spurious group differences, as we have shown with an example of the RL model with forgetting processes. We demonstrated that the lack of forgetting process, which differs between two groups, leads to a larger regression coefficient of RPE for the group with a small forgetting rate compared to the group with a large forgetting rate. Given that individuals with depression tend to have larger forgetting rates [32], the inconsistent results observed in blunted RPE responses in individuals with depression may be attributed to this model-misspecification. Indeed, these remain speculative, but our results call for further examination of previous results with a refined model parameter estimation method and broad types of RL models (e.g., with forgetting processes).

To avoid spurious effects due to the model-misspecification, the candidate model to be compared should include models incorporating potential components (e.g., a forgetting process) that can affect the latent-variable estimates. However, it is usually difficult to judge whether the current candidate models sufficiently include possible components that should be considered. One promising approach is to use a recurrent neural network (RNNs), which can flexibly learn to represent the arbitrary history dependence of choice from observed choice data [65, 66]. By comparing the statistical properties of the RNNs’ prediction and the candidate computational model’s prediction (i.e., how the choice depends on the choice and reward history), one can check whether there is any unmodeled component. Note that RNNs do not explicitly represent reward prediction error or other latent variables, and thus they cannot be used to construct model-based regressors. In addition, the best model may differ across subjects or groups, causing model-misspecification for a specific group of subjects. Piray et al. [59] addressed this issue by proposing a hierarchical Bayesian inference framework in which model fitting and model comparison are concurrently conducted. How this framework works for model-based fMRI remains elusive.

We have demonstrated that the design of the behavioral task (e.g., reward schedule and the number of trials) influences how the model misfit affects the results of model-based fMRI. As we have observed, the relation between the degree of the bias and reward schedule parameters is not monotonic. Thus, a suitable reward schedule that suppresses the spurious effect depends on the models and the property of the subjects’ behavior. One method to choose a task design is to observe how the bias arises by performing a model simulation generating the surrogate behavioral and neural data [1]. Some psychiatric and neuronal diseases are linked to adaptivity to changing contingency, which may further influence the results [67]. We also reported that as the number of trials increases, the spurious effect also increases. Thus, increasing the number of trials cannot solve the problem of spurious effects. Of course, using as many trials as possible is advantageous in that the parameter estimates become stable, and the true effect can be easily detected.

After we conducted the present study, we realized that Kumar et al. [26], who shows the blunted RPE response in unmedicated depression, reported that the effect size of group differences in RPE response remained unchanged even when using fit learning rates individually fit to the subjects, compared to when using a common learning rate (Fig S13C in [26]). This result indicates that diminished RPE responses in depression are not solely accounted for by the bias caused by misspecification of learning rates, at least in their data (but note that the effect size for punishment prediction error decreased when individual parameters were used; Fig S13D in [26]). On the other hand, as Kumar et al. did not include the forgetting process in their reinforcement learning model, there remains the possibility that the model-misspecification (e.g., lack of forgetting processes, which might differ in subjects with depression from healthy controls) can account for the diminished RPE responses in depression. A further consideration is required regarding the effect of model-misspecification on blunted RPE responses in depression.

In conclusion, appropriate model-fitting (including parameter estimation and model selection) is particularly important for model-based fMRI that focuses on group comparison and correlation analyses that seek neural or behavioral correlates of the severity of psychiatric symptoms or individual traits. Thus, the present study highlights the importance of model-fitting, especially in computational psychiatry. Our results also highlight the importance of exploring good models of computational processes underlying behavior and symptoms.

## Materials and methods

The basic procedures considered in this study are as follows. First, we simulate a ground-truth computational process of learning and the corresponding neural activity (e.g., BOLD signal) assumed to be observed. To achieve this, we assume specific reinforcement learning models and learning tasks such as classical conditioning tasks or probabilistic reward learning tasks. Neural activity is simulated by a linear model or a general linear model (GLM) with the true latent variable of the learning model as a regressor. Next, we simulate the standard model-based analysis of neural data: GLM with regressors constructed by latent variables with fitted model parameters, which in general deviate from the ground-truth values, are applied to neural data, and beta values (regression coefficients) were estimated. We assume multiple individuals with different true parameters. The beta values are compared between groups or individuals.

We want to evaluate how the mismatch between the fit and the true model influence beta values and the difference between groups. The evaluation is done either analytically or numerically depending on the problem: while a simple scenario for the RW model can be evaluated analytically, for the cases where an analytical calculation is infeasible (e.g., cases where within-group heterogeneity exists, and RL models with forgetting), we perform numerical simulations.

In the following, we first introduce the general GLM and the estimates of regression coefficients. Then we provide some analytic expressions for the statistics of beta values. Next, for each model (the RW model and the RL model with forgetting), simulation procedure and/or analytical results are described.

### General linear model (GLM)

In this paper, GLMs are used in two ways as in [21]: one is to model the generative processes of BOLD signal (used as a “true model”) and the other is to analyze such BOLD signal data, as in usual fMRI data analysis. GLMs assume that a single response variable (e.g., BOLD signal), *y*_*t*_, where *t* denotes the time point or trial, is explained in terms of a linear combination of the regressors and an error term. For descriptive purposes, we consider here a GLM with two regressors (predictors), *x*_*t*1_ and *x*_*t*2_, but our results can be applied to models with more than two regressors. We assume that the response variable and regressors are centered such that their means are zero. In the simulations, however, we did not mean-center the response variable and instead included the intercept term. This does not influence the estimates of regression coefficients of interest.

The GLM we consider here is written as follows:

$$\begin{array}{c} y_{t}=\beta_{1}x_{t1}+\beta_{2}x_{t2}+\epsilon_{t} \end{array}$$ (11)

where the *i*th regressor is denoted by *x*_*ti*_. *ϵ*_*t*_ is independent and identically distributed Gaussian random variable with mean zero and variance $\sigma_{\epsilon }^{2}$. With vector and matrix notations:

$$\begin{array}{c} Y=[\begin{array}{c} y_{1}\\ y_{2}\\ \vdots \\ y_{T} \end{array}],x_{1}=[\begin{array}{c} x_{11}\\ x_{21}\\ \vdots \\ x_{T1} \end{array}],x_{2}=[\begin{array}{c} x_{12}\\ x_{22}\\ \vdots \\ x_{T2} \end{array}],X=[\begin{array}{cc} x_{1} & x_{2} \end{array}],\beta =[\begin{array}{c} \beta_{1}\\ \beta_{2} \end{array}],\;\epsilon =[\begin{array}{c} \epsilon_{1}\\ \epsilon_{2}\\ \vdots \\ \epsilon_{T} \end{array}], \end{array}$$

GLM for data from *t* = 1 to *t* = *T* (from a single subject) are represented as

$$\begin{array}{c} Y=X\beta +\epsilon . \end{array}$$ (12)

By the method of ordinary least squares, we obtain the estimates of the regression coefficients as

$$\begin{array}{c} \hat{\beta }={\left(X^{\prime }X\right)}^{-1}X^{\prime }Y, \end{array}$$ (13)

where ⋅′ denotes the matrix transpose and ⋅^−1^ denotes the matrix inverse.

Next, we provide component-wise estimates for a case of two regressors. With the following quantities:

$$S(x_{1},x_{1})=x_{1}^{\prime }x_{1}=\sum_{t=1}^{T}x_{t1}^{2},$$ (14)

$$S(x_{1},x_{2})=x_{1}^{\prime }x_{2}=\sum_{t=1}^{T}x_{t1}x_{t2},$$ (15)

the estimate of *β*_1_ is expressed as

$$\begin{array}{c} {\hat{\beta }}_{1}=\frac{S\left(x_{2},x_{2}\right)S\left(x_{1},Y\right)-S\left(x_{1},x_{2}\right)S\left(x_{2},Y\right)}{S\left(x_{1},x_{1}\right)S\left(x_{2},x_{2}\right)-S{\left(x_{1},x_{2}\right)}^{2}}. \end{array}$$ (16)

When the covariance between regressors are zero, i.e., *S*(***x***_1_, ***x***_2_) = 0, this becomes

$$\begin{array}{c} {\hat{\beta }}_{1}=\frac{S(x_{1},Y)}{S(x_{1},x_{1})}. \end{array}$$ (17)

This corresponds to the beta value for ***x***_1_ when GLM includes only ***x***_1_ as a regressor. The estimate of *β*_2_ is similarly obtained.

#### Notes on assumptions of present study

Following Wilson & Niv [21], our simulations and analytical calculations treated latent variables as regressors for model-based fMRI (i.e., we performed simulation analysis in a trial space rather than in continuous experimental time). In real model-based fMRI, however, the regressors for BOLD signals are usually constructed by convolving the impulse sequence with a hemodynamic response function (HRF), where the height of impulses (stick functions) are parametrically modulated by the variable of interest (e.g., RPE) and the timing of impulse is usually set at stimulus onset (e.g., when a reward is presented). In S6 Text we examined the impact of ignoring the time course of hemodynamic responses, which had only minor effects on the result. This indicates that considering a raw latent variable (without convolution with hemodynamic response function) as a regressor is sufficient for our purpose.

In addition to mean-centered all model-based regressors, Wilson & Niv [21] normalized (z-scored) regressors so that their standard deviation is 1. In S5 Text, we discuss the effect of normalization of regressors and BOLD signal. It turns out that the normalization of regressors do not influence the effect size of interest in this paper. Although the normalization of neural signals, *y*_*t*_, affects the results by weakening the effect size of group differences, this effect remains after the normalization.

It should also be noted that all the GLMs considered are assumed to be able to represent ground-truth data generating processes if ground-truth model parameters (e.g., learning rate) are used. Nevertheless, GLM2 shows higher goodness of fit to hypothetical BOLD signal compared to GLM1 because the GLM2 has the flexibility to fit noise due to the separate regressors. However, the flexibility of GLM2 does not induce any bias for the beta values, given that the noise symmetrically distributes around the prediction of the BOLD signal, as we have assumed.

### Statistics of estimates of regression coefficients

Next, we consider statistics (mean and variance) of the estimates of regression coefficients, $\hat{\beta }$, under the assumption that *Y* is generated from GLM with ground-truth regressor $X^{*}=\left[x_{1}^{*}\;x_{2}^{*}\right]$ where *i*th (ground truth) regressor is denoted by $x_{i}^{*}={\left[x_{1i}^{*}\;x_{2i}^{*}\cdots x_{Ti}^{*}\right]}^{\prime }$.

From Eqs 12 and 13, we have

$$\hat{\beta }={\left(X^{\prime }X\right)}^{-1}X^{\prime }\left(X^{*}\beta +\epsilon \right).$$ (18)

Taking the expectation over noise ***ϵ***, noting the mean of noise, *ϵ*, is zero (***ϵ*** = **0**), we get

$$\text{E}[\hat{\beta }]={\left(X^{\prime }X\right)}^{-1}X^{\prime }X^{*}\beta .$$ (19)

The analytical expressions of regression coefficients obtained in Wilson & Niv [21] correspond to this expected value. Note that in our model-based fMRI context, the expectation is taken over different realizations of noise in the BOLD signal, rather than over different realizations of reward sequences or RPE (when we fixed the reward sequence, RPE and values were also fixed).

For a case of two regressors (see the Section 3 of S1 Text for details), we obtain

$$\begin{array}{cc} \text{E}[{\hat{\beta }}_{1}]= & [(S\left(x_{2},x_{2}\right)S\left(x_{1},x_{1}^{*}\right)-S\left(x_{1},x_{2}\right)S\left(x_{1}^{*},x_{2}\right))\beta_{1}\\ & +(S\left(x_{2},x_{2}\right)S\left(x_{1},x_{2}^{*}\right)-S\left(x_{1},x_{2}\right)S\left(x_{2}^{*},x_{2}\right))\beta_{2}]\\ & /(S\left(x_{1},x_{1}\right)S\left(x_{2},x_{2}\right)-S{\left(x_{1},x_{2}\right)}^{2}), \end{array}$$ (20)

$$\begin{array}{cc} \text{E}[{\hat{\beta }}_{2}]= & [(S\left(x_{1},x_{1}\right)S\left(x_{1}^{*},x_{2}\right)-S\left(x_{1},x_{2}\right)S\left(x_{1},x_{1}^{*}\right))\beta_{1}\\ & +(S\left(x_{1},x_{1}\right)S\left(x_{2},x_{2}^{*}\right)-S\left(x_{1},x_{2}\right)S\left(x_{1},x_{2}^{*}\right))\beta_{2}]\\ & /(S\left(x_{1},x_{1}\right)S\left(x_{2},x_{2}\right)-S{\left(x_{1},x_{2}\right)}^{2}). \end{array}$$ (21)

For the GLM with only a single regressor ***x***_1_, by setting $S(x_{1},x_{2}^{*})=0$ in Eq 20, we obtain

$$\begin{array}{cc} \text{E}[{\hat{\beta }}_{1}] & =\frac{S(x_{1},x_{1}^{*})}{S(x_{1},x_{1})}\beta_{1}\\ & =\frac{S(x_{1},x_{1}^{*})}{\sqrt{S(x_{1},x_{1})}\sqrt{S(x_{1}^{*},x_{1}^{*})}}\beta_{1}\frac{\sqrt{S(x_{1}^{*},x_{1}^{*})}}{\sqrt{S(x_{1},x_{1})}}\\ & =\text{Cor}\left(x_{1},x_{1}^{*}\right)\frac{\sqrt{S(x_{1}^{*},x_{1}^{*})}}{\sqrt{S(x_{1},x_{1})}}\beta_{1}, \end{array}$$ (22)

where $\text{Cor}(x_{1},x_{1}^{*})$ denotes the correlation coefficient between ***x***_1_ and $x_{1}^{*}$. This is equivalent with the analytical result in Wilson & Niv [21] (Eq 2 in [21]).

Next, the variance and covariance of regression coefficients need to be evaluated to obtain the effect size of group-level statistics. The variance-covariance matrix is calculated as

$$\begin{array}{cc} \text{Cov}[\hat{\beta }] & ={\left(X^{\prime }X\right)}^{-1}X^{\prime }\text{Var}\left[Y\right]X{\left(X^{\prime }X\right)}^{-1}\\ & ={\left(X^{\prime }X\right)}^{-1}X^{\prime }\left(\sigma_{\epsilon }^{2}I\right)X{\left(X^{\prime }X\right)}^{-1}\\ & =\sigma_{\epsilon }^{2}{\left(X^{\prime }X\right)}^{-1}X^{\prime }X{\left(X^{\prime }X\right)}^{-1}\\ & =\sigma_{\epsilon }^{2}{\left(X^{\prime }X\right)}^{-1}, \end{array}$$ (23)

where ***I*** denotes the identity matrix with the size being the number of regressors. For a case with two regressors, we have

$$\text{Cov}[\hat{\beta }]=\frac{\sigma_{\epsilon }^{2}}{S\left(x_{1},x_{1}\right)S\left(x_{2},x_{2}\right)-S{\left(x_{1},x_{2}\right)}^{2}}[\begin{array}{cc} S(x_{2},x_{2}) & -S(x_{1},x_{2})\\ -S(x_{1},x_{2}) & S(x_{1},x_{1}) \end{array}].$$ (24)

From this, the variance of ${\hat{\beta }}_{1}$ is obtained as follows

$$\text{Var}[{\hat{\beta }}_{1}]=\frac{\sigma_{\epsilon }^{2}S\left(x_{2},x_{2}\right)}{S\left(x_{1},x_{1}\right)S\left(x_{2},x_{2}\right)-S{\left(x_{1},x_{2}\right)}^{2}}$$ (25)

$$\begin{array}{cc} & =\frac{\sigma_{\epsilon }^{2}}{S\left(x_{1},x_{1}\right)(1-\text{Cor}{\left(x_{1},x_{2}\right)}^{2})}. \end{array}$$ (26)

For the GLM with only a single regressor the variance of ${\hat{\beta }}_{1}$ is

$$\text{Var}[{\hat{\beta }}_{1}]=\frac{\sigma_{\epsilon }^{2}}{S(x_{1},x_{1})}.$$ (27)

In common fMRI data analysis, a beta value (denoted simply here as *β*) fitted to each subject (first-level analysis) is treated as a random variable and subject to a one-sample t-test (second-level analysis) with the null hypothesis that the population mean of $\hat{\beta }$ is zero. This test is based on the test statistic:

$$\begin{array}{c} t_{1}=\frac{\bar{\hat{\beta }}}{s(\hat{\beta })}\sqrt{n}, \end{array}$$ (28)

where $\bar{\hat{\beta }}$ and $s(\hat{\beta })$ are the sample mean and sample standard deviation of $\hat{\beta }$, respectively. *n* is the number of subjects included in the group.

Under the alternative hypothesis: population mean of $\hat{\beta }$ is not equal to zero, *t*_1_ obeys a noncentral t-distribution with *n* − 1 degrees of freedom with the noncentrality parameter

$$\begin{array}{c} \lambda =d_{1}\sqrt{n}. \end{array}$$ (29)

where *d*_1_ denotes the group-level effect size of *β* (for a single group) given by Eq 8. The larger λ is, the larger the statistical power. Thus, the effect size, *d*_1_, can be used as a measure of statistical power.

When the regression coefficients of two groups are compared using a two-sample t-test, the corresponding effect size, *d*_2_, is given by Eq 7.

The test statistic used for the two-sample t-test (assuming homogeneity of population variance) is

$$t_{12}=\frac{{\bar{\hat{\beta }}}^{\left(1\right)}-{\bar{\hat{\beta }}}^{\left(2\right)}}{s^{*}}\sqrt{\frac{n_{1}n_{2}}{n_{1}+n_{2}}},$$ (30)

where *n*_1_ and *n*_2_ are the number of subjects included in the 1st and 2nd group, respectively, and s* denotes the estimates of (common) population standard deviation given by

$$s^{*}=\sqrt{\frac{\left(n_{1}-1\right)s^{2}\left({\hat{\beta }}^{\left(1\right)}\right)+\left(n_{2}-1\right)s^{2}\left({\hat{\beta }}^{\left(2\right)}\right)}{n_{1}+n_{2}-2}(\frac{1}{n_{1}}+\frac{1}{n_{2}})}.$$ (31)

The test statistic *t*_12_ obeys a noncentral t-distribution with *n*_1_ + *n*_2_ − 2 degrees of freedom and the noncentrality parameter given by

$$\begin{array}{c} \lambda =d_{2}\sqrt{\frac{n_{1}n_{2}}{n_{1}+n_{2}}}. \end{array}$$ (32)

Strictly speaking, this holds when the population variances of two beta values are equal (i.e., $\text{Var}\left[{\hat{\beta }}^{\left(1\right)}\right]=\text{Var}\left[{\hat{\beta }}^{\left(2\right)}\right]$). Using these facts, the statistical power can be obtained using the R package “pwr.”

### Rescorla-Wagner (RW) model

As a concrete example of computational models, we consider an RW model following [21]. We here repeat the equations for RW model in Results section (Eqs 1 and 9) with the reward sensitivity parameter, *ρ*:

$$V_{t+1}=V_{t}+\alpha \delta_{t},$$

$$\delta_{t}=\rho r_{t}-V_{t}.$$

The analytic results for the model with no reward sensitivity parameter are obtained by setting $\rho^{*}=\hat{\rho }=1$.

Below we present the analytical result for the statistics of beta values. In the case where the reward probability is fixed to *p*_*r*_, the quantities appearing in Eqs 20 and 21 are approximately obtained as follows, by utilizing the analytical method in [21] (see S1 Text for detailed derivation):

$$S(\hat{\delta },\hat{\delta })=S\left(-\hat{V},-\hat{V}\right)=T\cdot \frac{2}{2-\hat{\alpha }}p_{r}\left(1-p_{r}\right){\hat{\rho }}^{2},$$ (33)

$$S(\delta ,\hat{\delta })=S\left(-V,-\hat{V}\right)=T\cdot \frac{\alpha^{*}+\hat{\alpha }}{\alpha^{*}+\hat{\alpha }-\alpha^{*}\hat{\alpha }}p_{r}\left(1-p_{r}\right)\hat{\rho }\rho^{*},$$ (34)

$$S(\hat{\delta },r)=T\cdot p_{r}\left(1-p_{r}\right)\hat{\rho },$$ (35)

$$S(\delta ,r)=T\cdot p_{r}\left(1-p_{r}\right)\rho^{*},$$ (36)

$$S(r,r)=T\cdot p_{r}\left(1-p_{r}\right),$$ (37)

$$S(r,-V)=S(r,-\hat{V})=0.$$ (38)

By substituting these expressions into the statistics of regression coefficients (Eqs 20, 21 and 22), we can obtain the expected value and variance of the beta values.

#### GLM 1

For GLM1 (RPE, $\hat{\delta }$ is a sole regressor), from Eqs 22 and 27, (We replace ***x***_1_ with RPE calculated with fit parameters, denoted by $\hat{\delta }$, and $x_{1}^{*}$ with ground-truth RPE signal denoted by ***δ***),

$$\text{E}[{\hat{\beta }}_{\delta }]=\frac{\left(2-\hat{\alpha }\right)\left(\alpha^{*}+\hat{\alpha }\right)}{2(\alpha^{*}+\hat{\alpha }-\alpha^{*}\hat{\alpha })}\beta_{\delta }\cdot \frac{\rho^{*}}{\hat{\rho }},$$ (39)

$$\text{Var}[{\hat{\beta }}_{\delta }]=\frac{\left(2-\hat{\alpha }\right)\sigma_{\epsilon }^{2}}{2Tp_{r}\left(1-p_{r}\right)}\cdot \frac{1}{{\hat{\rho }}^{2}}.$$ (40)

#### GLM 2

For GLM2, where the reward *r* and negative value −*V* are the regressors, we replace both ***x***_1_ and $x_{1}^{*}$ with ***r***, ***x***_2_ with $-\hat{V}$, and $x_{2}^{*}$ with −***V***. We assume $x_{2}=x_{2}^{*}$ because the reward sequence is assumed to be observed without error. For regression coefficient *β*_*NV*_ (for negative value, −*V*) in GLM2,

$$\text{E}[{\hat{\beta }}_{NV}]=\frac{\alpha^{*}\left(2-\hat{\alpha }\right)}{\alpha^{*}+\hat{\alpha }-\alpha^{*}\hat{\alpha }}\beta_{\delta }\cdot \frac{\rho^{*}}{\hat{\rho }},$$ (41)

$$\text{Var}[{\hat{\beta }}_{NV}]=\frac{\left(2-\hat{\alpha }\right)\sigma_{\epsilon }^{2}}{T\hat{\alpha }p_{r}\left(1-p_{r}\right)}\cdot \frac{1}{{\hat{\rho }}^{2}}.$$ (42)

For the regression coefficient for reward, ${\hat{\beta }}_{r}$,

$$\text{E}[{\hat{\beta }}_{r}]=\beta_{r}\cdot \rho^{*},$$ (43)

$$\text{Var}[{\hat{\beta }}_{r}]=\frac{\sigma_{\epsilon }^{2}}{Tp_{r}\left(1-p_{r}\right)}.$$ (44)

#### GLM 2′

For GLM 2′, where the regressors are RPE and reward,

$$\text{E}[{\hat{\beta }}_{\delta }]=\frac{\alpha^{*}\left(2-\hat{\alpha }\right)}{\alpha^{*}+\hat{\alpha }-\alpha^{*}\hat{\alpha }}\beta_{\delta }\cdot \frac{\rho^{*}}{\hat{\rho }},$$ (45)

$$\text{Var}[{\hat{\beta }}_{\delta }]=\frac{\left(2-\hat{\alpha }\right)\sigma_{\epsilon }^{2}}{T\hat{\alpha }p_{r}\left(1-p_{r}\right)}\cdot \frac{1}{{\hat{\rho }}^{2}}.$$ (46)

Note that these are identical as ${\hat{\beta }}_{NV}$ in GLM2. For the regression coefficient for reward, ${\hat{\beta }}_{r}$ in GLM2′,

$$\text{E}[{\hat{\beta }}_{r}]=\frac{\hat{\alpha }-\alpha^{*}}{\alpha^{*}+\hat{\alpha }-\alpha^{*}\hat{\alpha }}\beta_{\delta }\cdot \frac{\rho^{*}}{\hat{\rho }}+\beta_{r}\cdot \rho^{*},$$ (47)

$$\text{Var}[{\hat{\beta }}_{r}]=\frac{2\sigma_{\epsilon }^{2}}{T\hat{\alpha }p_{r}\left(1-p_{r}\right)}.$$ (48)

From the first equation, we notice that when $\alpha^{*}\neq \hat{\alpha }$, the estimate of *β*_*r*_ is influenced by the true value of *β*_*δ*_.

#### Simulation procedure

For results reported in Figs 1 and 2, we generated reward sequences in a classical conditioning paradigm where the reward probability was *p*_*r*_ = 0.4. The task for each hypothetical subject contained 100 trials. Specifically, the reward sequence was generated by shuffling an array containing 40 reward and 60 non-reward trials. As in [42], the same realization of the reward sequence was used for all hypothetical subjects. We set parameters of GLM for generating hypothetical neural signals to *σ*_*ϵ*_ = 0.5, *β*_*V*_ = 1.0 (for the simulation in Fig 1), and *β*_*δ*_ = 1.0 (for other simulations).

For the section “The effect of within-group heterogeneity” (Fig 5), true learning rates were drawn from a (truncated) Gaussian distribution with a mean of 0.2 for Low-L group and 0.4 for High-L group, and s.d. varied between 0.05-0.5 with a step size of 0.05. Sample values of less than 0 were replaced with 0.0, and sample values larger than 1 were replaced with 1.0. We assumed that the common parameter, $\hat{\alpha }=0.3$, is used for both groups to derive estimated RPE signal, $\hat{\delta }$, and negative value, $-\hat{V}$. To obtain stable estimates of the effect size, we simulated *N* = 5000 hypothetical participants for each group.

### Reinforcement learning with forgetting

In simulations examining the effects of model-misspecification, we used the RL model with forgetting as a true model. In the model fitting to the synthesized dataset, the standard RL model without forgetting processes and the RL model with forgetting, which had the same model structure as the true model, were used. In these models, the action value for the chosen option *i*, *V*(*i*) was updated similarly to the RW model (Eqs 1 and 2). For the unchosen option *j* (*j* ≠ *i*), the action value, *V*(*j*), was updated in the RL model with forgetting as follows (we repeat Eq 10, with the index of actions):

$$V_{t+1}\left(j\right)=V_{t}\left(j\right)+\phi \left(\mu -V_{t}\left(j\right)\right),$$ (49)

where *ϕ* is the forgetting rate and *μ* is the default value [32, 33]. In the standard RL model, the action value of the unchosen option was not updated (this corresponds to *ϕ* = 0). The initial action values were set to the default value (i.e., *V*_1_(1) = *V*_1_(2) = *μ*). Throughout the simulation, we set the default value as *μ* = 0.5 in both the true and fit models.

Based on the set of the action values, the model assigns the probability of choosing the option *i* using the soft-max function:

$$\begin{array}{c} P\left(\text{choosing}\;\text{option}\;i\right)=\frac{1}{1+\text{exp}(-\beta [V_{t}\left(i\right)-V_{t}\left(j\right)])}, \end{array}$$ (50)

where *j* indicates another option and *β* is termed the inverse temperature parameter (not to be confused with regression coefficients), which determines the sensitivity of the choice probabilities to the differences in action values.

#### Simulation procedure

The details of the simulations are as follows. First, we simulated repeated choices using the RL model with the forgetting process in a probabilistic reversal learning task where one of the options was associated with a high reward probability of 0.8, while the other option was associated with a low reward probability of 0.2. With the probability for the chosen option, the reward was given (*r*_*t*_ = 1); otherwise, no reward was given (*r*_*t*_ = 0). After 90 trials, the contingencies of the two stimuli were reversed. In total, data for 180 choice trials were generated for each hypothetical subject. We employed the probabilistic reversal learning task because the reversal of the reward contingency emphasizes the effect of forgetting [31]. In supporting material (S2 Fig), we performed simulations with various number of reversals.

Tasks with different reward sequences were generated and 100 simulations were run; half of them used the RL model with large involvement of the forgetting component (*ϕ* = 0.4) as a true model. The data were regarded as those from the hypothetical patient group (High-F group). The other half used the RL model with little involvement from the forgetting component (*ϕ* = 0.05) as a true model, and the data were regarded as those from the hypothetical control subjects (Low-F group). Common true parameters for all cases were *α* = 0.5, and *β* = 4.0. We generated target neural signals reflecting RPE, using Eq 4 where *β*_*δ*_ = 1 and *σ*_*ϵ*_ = 0.5. To estimate the RL model parameters, maximum likelihood estimation (MLE), which searches a parameter set that maximizes the log-likelihood for all choices was separately performed for data from each hypothetical subject. These estimations were performed using the rsolnp 1.16 package, which implements the augmented Lagrange multiplier method with an SQP interior algorithm [68]. To facilitate finding the global optimum solution, the algorithms were run 10 times; each run was initiated from a random initial value, and the parameter set that provided the lowest negative log likelihood was selected.

### Software and code availability

For all simulations, analysis, and plots, we used R, version 3.2.0 (http://cran.us.r-project.org). All codes required to reproduce the results presented in this paper are available on https://github.com/kkatahira/model-based_fMRI (Github).

## Supporting information

S1 Text Derivation of the statistics in Rescorla-Wagner model and GLMs. (PDF)

S2 Text Effects of reward sensitivity. (PDF)

S3 Text Dependence of effect size on the combinations of the ground-truth learning rates. (PDF)

S4 Text Effects of reward contingency. (PDF)

S5 Text Effects of normalization (z-scoring) in GLMs. (PDF)

S6 Text Impacts of ignoring time course of BOLD signal. (PDF)

S1 FigChecking the validity of analytical expressions for the effect size of group comparison for different number of trials, *T*.(A) For beta value of RPE in GLM1. (B) For beta value of negative value, −*V*, in GLM2. Gray dots represents the results of single simulation run. The number of trials was varied within 3, 10, 25, and from 50 to 500 with the step size 50. Except for that, the simulation setting is the same with those for Fig 2: We assumed the true learning rate of Low-L group (patient group) was *α* = 0.2, and that of High-L group (healthy control) was *α* = 0.4. The fit learning rate was $\hat{\alpha }=0.3$.(PNG)

S2 Fig Effect of the number of reversals in the simulation of model-misspecification (RL model with forgetting). The effect size (Cohen’s d) for between-group difference of beta values are plotted as a function of the number of reversals of reward contingency. We simulated different frequencies of reward-contingency reversal (0, 1, 2, 5, 8, 17, or 35). For each reversal condition, we ran 100 simulations by producing anew reward history. Settings other than the reward contingency were the same with Fig 7 (“Effect of model-misspecification” section). This panel shows the mean effect size in each condition with error bars representing the standard error. For beta values of RPE in GLM1 and negative value in GLM2, the higher the frequency of the reversal, the larger the effect size. This reflects that the effect of the forgetting component has a larger effect on choice behavior when reversals are frequent. However, too many reversals (35 reversals in the panel) diminished effect size by ambiguating group differences in behavioral choices.
(PNG)

## References

[1] Wilson RC, Collins AG. Ten simple rules for the computational modeling of behavioral data. eLife. 2019;8:e49547 10.7554/eLife.49547 31769410 PMC6879303

[2] O’Doherty JP, Dayan P, Friston K, Critchley H, Dolan RJ. Temporal difference models and reward-related learning in the human brain. Neuron. 2003;38(2):329–337. 10.1016/S0896-6273(03)00169-7 12718865

[3] Tanaka SC, Doya K, Okada G, Ueda K, Okamoto Y, Yamawaki S. Prediction of immediate and future rewards differentially recruits cortico-basal ganglia loops. Nature Neuroscience. 2004;7(8):887–893. 10.1038/nn1279 15235607

[4] O’Doherty JP. Reward representations and reward-related learning in the human brain: insights from neuroimaging. Current Opinion in Neurobiology. 2004;14(6):769–776. 10.1016/j.conb.2004.10.016 15582382

[5] O’Doherty JP, Hampton A, Kim H. Model-Based fMRI and Its Application to Reward Learning and Decision Making. Annals of the New York Academy of sciences. 2007;1104(1):35–53. 10.1196/annals.1390.022 17416921

[6] Gläscher JP, O’Doherty JP. Model-based approaches to neuroimaging: combining reinforcement learning theory with fMRI data. Wiley Interdisciplinary Reviews: Cognitive Science. 2010;1(4):501–510. 26271497 10.1002/wcs.57

[7] O’Doherty J, Critchley H, Deichmann R, Dolan RJ. Dissociating valence of outcome from behavioral control in human orbital and ventral prefrontal cortices. Journal of Neuroscience. 2003;23(21):7931–7939. 10.1523/JNEUROSCI.23-21-07931.2003 12944524 PMC6740603

[8] Pessiglione M, Seymour B, Flandin G, Dolan RJ, Frith CD. Dopamine-dependent prediction errors underpin reward-seeking behaviour in humans. Nature. 2006;442(7106):1042–1045. 10.1038/nature05051 16929307 PMC2636869

[9] Daw N, O’Doherty JP, Dayan P, Seymour B, Dolan RJ. Cortical substrates for exploratory decisions in humans. Nature. 2006;441(7095):876–879. 10.1038/nature04766 16778890 PMC2635947

[10] Cavanagh JF, Frank MJ, Klein TJ, Allen JJ. Frontal theta links prediction errors to behavioral adaptation in reinforcement learning. NeuroImage. 2010;49(4):3198–3209. 10.1016/j.neuroimage.2009.11.080 19969093 PMC2818688

[11] Ichikawa N, Siegle GJ, Dombrovski A, Ohira H. Subjective and model-estimated reward prediction: Association with the feedback-related negativity (FRN) and reward prediction error in a reinforcement learning task. International Journal of Psychophysiology. 2010;78(3):273–283. 10.1016/j.ijpsycho.2010.09.001 20858518 PMC3150511

[12] Bai Y, Katahira K, Ohira H. Valence-separated representation of reward prediction error in feedback-related negativity and positivity. NeuroReport. 2015;26(3):157–162. 10.1097/WNR.0000000000000318 25634316

[13] Samejima K, Ueda Y, Doya K, Kimura M. Representation of action-specific reward values in the striatum. Science. 2005;310(5752):1337–1340. 10.1126/science.1115270 16311337

[14] Ito M, Doya K. Validation of decision-making models and analysis of decision variables in the rat basal ganglia. Journal of Neuroscience. 2009;29(31):9861 10.1523/JNEUROSCI.6157-08.2009 19657038 PMC6666589

[15] Nassar MR, Rumsey KM, Wilson RC, Parikh K, Heasly B, Gold JI. Rational regulation of learning dynamics by pupil-linked arousal systems. Nature Neuroscience. 2012;15(7):1040 10.1038/nn.3130 22660479 PMC3386464

[16] Dombrovski AY, Hallquist MN, Brown VM, Wilson J, Szanto K. Value-Based Choice, Contingency Learning, and Suicidal Behavior in Mid-and Late-Life Depression. Biological Psychiatry. 2019;85(6):506–516. 10.1016/j.biopsych.2018.10.006 30502081 PMC6380943

[17] Nassar MR, Gold JI. A healthy fear of the unknown: perspectives on the interpretation of parameter fits from computational models in neuroscience. PLoS Computational Biology. 2013;9(4):e1003015 10.1371/journal.pcbi.1003015 23592963 PMC3617224

[18] Nassar MR, Frank MJ. Taming the beast: extracting generalizable knowledge from computational models of cognition. Current Opinion in Behavioral Sciences. 2016;11:49–54. 10.1016/j.cobeha.2016.04.003 27574699 PMC5001502

[19] Toyama A, Katahira K, Ohira H. Biases in estimating the balance between model-free and model-based learning systems due to model misspecification. Journal of Mathematical Psychology. 2019;91:88–102. 10.1016/j.jmp.2019.03.007

[20] Katahira K. The statistical structures of reinforcement learning with asymmetric value updates. Journal of Mathematical Psychology. 2018;87:31–45. 10.1016/j.jmp.2018.09.002

[21] Wilson RC, Niv Y. Is Model Fitting Necessary for Model-Based fMRI? PLoS Computational Biology. 2015;11(6):e1004237 10.1371/journal.pcbi.1004237 26086934 PMC4472514

[22] Kumar P, Waiter G, Ahearn T, Milders M, Reid I, Steele J. Abnormal temporal difference reward-learning signals in major depression. Brain. 2008;131(8):2084–2093. 10.1093/brain/awn136 18579575

[23] Gradin VB, Kumar P, Waiter G, Ahearn T, Stickle C, Milders M, et al Expected value and prediction error abnormalities in depression and schizophrenia. Brain. 2011;134(6):1751–1764. 10.1093/brain/awr059 21482548

[24] Murray G, Corlett P, Clark L, Pessiglione M, Blackwell A, Honey G, et al Substantia nigra/ventral tegmental reward prediction error disruption in psychosis. Molecular Psychiatry. 2008;13(3):267–276. 10.1038/sj.mp.4002058 17684497 PMC2564111

[25] Ross MC, Lenow JK, Kilts CD, Cisler JM. Altered neural encoding of prediction errors in assault-related posttraumatic stress disorder. Journal of Psychiatric Research. 2018;103(February):83–90. 10.1016/j.jpsychires.2018.05.008 29783079 PMC6008230

[26] Kumar P, Goer F, Murray L, Dillon DG, Beltzer ML, Cohen AL, et al Impaired reward prediction error encoding and striatal-midbrain connectivity in depression. Neuropsychopharmacology. 2018;43(7):1581–1588. 10.1038/s41386-018-0032-x 29540863 PMC5983542

[27] Rutledge RB, Moutoussis M, Smittenaar P, Zeidman P, Taylor T, Hrynkiewicz L, et al Association of neural and emotional impacts of reward prediction errors with major depression. JAMA Psychiatry. 2017;74(8):790–797. 10.1001/jamapsychiatry.2017.1713 28678984 PMC5710549

[28] Rothkirch M, Tonn J, Köhler S, Sterzer P. Neural mechanisms of reinforcement learning in unmedicated patients with major depressive disorder. Brain. 2017;140(4):1147–1157. 10.1093/brain/awx025 28334960

[29] White SF, Geraci M, Lewis E, Leshin J, Teng C, Averbeck B, et al Prediction error representation in individuals with generalized anxiety disorder during passive avoidance. American Journal of Psychiatry. 2017;174(2):110–117. 10.1176/appi.ajp.2016.15111410 27631963 PMC5572647

[30] Daw N. Trial-by-trial data analysis using computational models. Decision Making, Affect, and Learning: Attention and Performance XXIII. 2011;23:1.

[31] Katahira K, Yuki S, Okanoya K. Model-based estimation of subjective values using choice tasks with probabilistic feedback. Journal of Mathematical Psychology. 2017;79:29–43. 10.1016/j.jmp.2017.05.005

[32] Toyama A, Katahira K, Ohira H. Reinforcement learning with parsimonious computation and a forgetting process. Frontiers in Human Neuroscience. 2019;13 10.3389/fnhum.2019.00153 31143107 PMC6520826

[33] Toyama A, Katahira K, Ohira H. A simple computational algorithm of model-based choice preference. Cognitive, Affective, & Behavioral Neuroscience. 2017;17(4):764–783. 10.3758/s13415-017-0511-2 28573384

[34] Behrens TE, Hunt LT, Woolrich MW, Rushworth MF. Associative learning of social value. Nature. 2008;456(7219):245–249. 10.1038/nature07538 19005555 PMC2605577

[35] Niv Y, Edlund JA, Dayan P, O’Doherty JP. Neural Prediction Errors Reveal a Risk-Sensitive Reinforcement-Learning Process in the Human Brain. Journal of Neuroscience. 2012;32(2):551–562. 10.1523/JNEUROSCI.5498-10.2012 22238090 PMC6621075

[36] Rescorla RA, Wagner AR. A theory of Pavlovian conditioning: Variations in the effectiveness of reinforcement and nonreinforcement. Classical conditioning II: Current research and theory. 1972;2:64–99.

[37] Bray S, O’Doherty J. Neural coding of reward-prediction error signals during classical conditioning with attractive faces. Journal of Neurophysiology. 2007;97(4):3036–3045. 10.1152/jn.01211.2006 17303809

[38] Bakker JM, Goossens L, Kumar P, Lange IM, Michielse S, Schruers K, et al From laboratory to life: associating brain reward processing with real-life motivated behaviour and symptoms of depression in non-help-seeking young adults. Psychological Medicine. 2019;49(14):2441–2451. 10.1017/S0033291718003446 30488820 PMC6541542

[39] Chase H, Frank M, Michael A, Bullmore E, Sahakian B, Robbins T. Approach and avoidance learning in patients with major depression and healthy controls: relation to anhedonia. Psychological Medicine. 2010;40(3):433–440. 10.1017/S0033291709990468 19607754

[40] Chen C, Takahashi T, Nakagawa S, Inoue T, Kusumi I. Reinforcement learning in depression: a review of computational research. Neuroscience & Biobehavioral Reviews. 2015;55:247–267. 10.1016/j.neubiorev.2015.05.005 25979140

[41] Robinson OJ, Chase HW. Learning and choice in mood disorders: searching for the computational parameters of anhedonia. Computational Psychiatry. 2017;1:208–233. 10.1162/CPSY_a_00009 29400358 PMC5796642

[42] Li J, Schiller D, Schoenbaum G, Phelps EA, Daw N. Differential roles of human striatum and amygdala in associative learning. Nature Neuroscience. 2011;14(10):1250–1252. 10.1038/nn.2904 21909088 PMC3268261

[43] Valentin VV, O’Doherty JP. Overlapping prediction errors in dorsal striatum during instrumental learning with juice and money reward in the human brain. Journal of Neurophysiology. 2009;102(6):3384–3391. 10.1152/jn.91195.2008 19793875

[44] Nagase AM, Onoda K, Foo JC, Haji T, Akaishi R, Yamaguchi S, et al Neural mechanisms for adaptive learned avoidance of mental effort. Journal of Neuroscience. 2018;38(10):2631–2651. 10.1523/JNEUROSCI.1995-17.2018 29431647 PMC6705903

[45] Gold BP, Mas-Herrero E, Zeighami Y, Benovoy M, Dagher A, Zatorre RJ. Musical reward prediction errors engage the nucleus accumbens and motivate learning. Proceedings of the National Academy of Sciences. 2019;116(8):3310–3315. 10.1073/pnas.1809855116 30728301 PMC6386687

[46] Mumford JA, Poline JB, Poldrack RA. Orthogonalization of regressors in fMRI models. PloS one. 2015;10(4). 10.1371/journal.pone.0126255 25919488 PMC4412813

[47] Huys QJ, Pizzagalli DA, Bogdan R, Dayan P. Mapping anhedonia onto reinforcement learning: a behavioural meta-analysis. Biol Mood Anxiety Disord. 2013;3(1):12 10.1186/2045-5380-3-12 23782813 PMC3701611

[48] Kunisato Y, Okamoto Y, Ueda K, Onoda K, Okada G, Yoshimura S, et al Effects of depression on reward-based decision making and variability of action in probabilistic learning. Journal of Behavior Therapy and Experimental Psychiatry. 2012;43(4):1088–94. 10.1016/j.jbtep.2012.05.007 22721601

[49] Katahira K. The relation between reinforcement learning parameters and the influence of reinforcement history on choice behavior. Journal of Mathematical Psychology. 2015;66:59–69. 10.1016/j.jmp.2015.03.006

[50] Robbins TW, Gillan CM, Smith DG, de Wit S, Ersche KD. Neurocognitive endophenotypes of impulsivity and compulsivity: towards dimensional psychiatry. Trends in Cognitive Sciences. 2012;16(1):81–91. 10.1016/j.tics.2011.11.009 22155014

[51] Hägele C, Schlagenhauf F, Rapp M, Sterzer P, Beck A, Bermpohl F, et al Dimensional psychiatry: reward dysfunction and depressive mood across psychiatric disorders. Psychopharmacology. 2015;232(2):331–341. 10.1007/s00213-014-3662-7 24973896 PMC4297301

[52] MacCallum RC, Zhang S, Preacher KJ, Rucker DD. On the practice of dichotomization of quantitative variables. Psychological Methods. 2002;7(1):19–40. 10.1037/1082-989X.7.1.19 11928888

[53] Altman DG, Royston P. The cost of dichotomising continuous variables. British Medical Journal. 2006;332(7549):1080 10.1136/bmj.332.7549.1080 16675816 PMC1458573

[54] Katahira K, Yamashita Y. A theoretical framework for evaluating psychiatric research strategies. Computational Psychiatry. 2017;1:184–207. 10.1162/CPSY_a_00008 30090858 PMC6067825

[55] Kato A, Morita K. Forgetting in reinforcement learning links sustained dopamine signals to motivation. PLoS Computational Biology. 2016;12(10):e1005145 10.1371/journal.pcbi.1005145 27736881 PMC5063413

[56] Ahn WY, Krawitz A, Kim W, Busemeyer JR, Brown JW. A model-based fMRI analysis with hierarchical Bayesian parameter estimation. Journal of Neuroscience, Psychology, and Economics. 2011;4(2):95 10.1037/a0020684 23795233 PMC3686299

[57] Katahira K. How hierarchical models improve point estimates of model parameters at the individual level. Journal of Mathematical Psychology. 2016;73:37–58. 10.1016/j.jmp.2016.03.007

[58] Brown VM, Chen J, Gillan CM, Price RB. Improving the reliability of computational analyses: Model-based planning and its relationship with compulsivity. Biological Psychiatry: Cognitive Neuroscience and Neuroimaging. 2020;5(6):601–609. 32249207 10.1016/j.bpsc.2019.12.019 PMC7286766

[59] Piray P, Dezfouli A, Heskes T, Frank MJ, Daw ND. Hierarchical Bayesian inference for concurrent model fitting and comparison for group studies. PLoS Computational Biology. 2019;15(6):e1007043 10.1371/journal.pcbi.1007043 31211783 PMC6581260

[60] Mkrtchian A, Aylward J, Dayan P, Roiser JP, Robinson OJ. Modeling avoidance in mood and anxiety disorders using reinforcement learning. Biological psychiatry. 2017;82(7):532–539. 10.1016/j.biopsych.2017.01.017 28343697 PMC5598542

[61] Valton V, Wise T, Robinson OJ. The Importance of Group Specification in Computational Modelling of Behaviour. PsyArXiv. 2020; 10.31234/osf.io/p7n3h.

[62] Boehm U, Marsman M, Matzke D, Wagenmakers EJ. On the importance of avoiding shortcuts in applying cognitive models to hierarchical data. Behavior Research Methods. 2018;50(4):1614–1631. 10.3758/s13428-018-1054-3 29949071 PMC6096647

[63] Sumiya M, Katahira K. Commentary: Altered learning under uncertainty in unmedicated mood and anxiety disorders. Frontiers in Human Neuroscience. 2020;14 10.3389/fnhum.2020.561770 33281579 PMC7691592

[64] Schönberg T, Daw N, Joel D, O’Doherty JP. Reinforcement learning signals in the human striatum distinguish learners from nonlearners during reward-based decision making. The Journal of Neuroscience. 2007;27(47):12860–12867. 10.1523/JNEUROSCI.2496-07.2007 18032658 PMC6673291

[65] Dezfouli A, Griffiths K, Ramos F, Dayan P, Balleine BW. Models that learn how humans learn: the case of decision-making and its disorders. PLoS Computational Biology. 2019;15(6):e1006903 10.1371/journal.pcbi.1006903 31185008 PMC6588260

[66] Dezfouli A, Ashtiani H, Ghattas O, Nock R, Dayan P, Ong CS. Disentangled behavioural representations. In: Advances in Neural Information Processing Systems; 2019. p. 2254–2263.

[67] Izquierdo A, Brigman JL, Radke AK, Rudebeck PH, Holmes A. The neural basis of reversal learning: an updated perspective. Neuroscience. 2017;345:12–26. 10.1016/j.neuroscience.2016.03.021 26979052 PMC5018909

[68] Ghalanos A, Theussl S. Rsolnp: general non-linear optimization using augmented Lagrange multiplier method, Version 1.15; 2011.

