# Supplementary material for: Revisiting the importance of model fitting for model-based fMRI: It does matter in computational psychiatry
Source: PLoS Comput Biol. 2021 Feb 9;17(2):e1008738. doi: 10.1371/journal.pcbi.1008738 (PMC7899379; doi:10.1371/journal.pcbi.1008738)
Supplement: S1 Text — (PDF) [file pcbi.1008738.s001.pdf]

Supplementary Material (S1 Text)
Revisiting the importance of model fitting for model-based fMRI: It does matter in computational psychiatry

Kentaro Katahira[1], Asako Toyama[1]

1 Department of Psychological and Cognitive Sciences, Nagoya University, Nagoya, Japan

1 Statistics in Rescorla-Wagner model

Here, we provide the derivation of the statistics of RPE and reward in the RW model. Our derivation basically follows Wilson & Niv [1], with minor modifications (e.g., including reward sensitivity parameter ρ).

The model equations are given by

$$V_{t+1} = V_t + \alpha\delta_t, \tag{1}$$

$$\delta_t = \rho r_t - V_t. \tag{2}$$

The initial value is set to zero, $V_1 = 0$. We assume that the reward probability is fixed to p_r over all trials.

The value sequence calculated with the parameter set $\{\alpha_i, \rho_i\}$ is denoted as V_{it} ($t = 1, ..., T$). Note that model equations Eqs 1, 2 can be rewritten as

$$V_{i(t+1)} = (1 - \alpha_i)V_{it} + \alpha_i \rho_i r_t$$

From this , for $t > 1$ (note that for $t = 1$, $V_{i1} = 0$), we have

$$V_{it} = \rho_i \sum_{a=1}^{t-1} \alpha_i (1 - \alpha_i)^{t-1-a} r_a. \tag{3}$$

Thus, the mean of the values over T trials, denoted by $\mu(\boldsymbol{V}_i)$ is

$$\mu(\boldsymbol{V}_i) = \frac{1}{T}\sum_{t=1}^{T} V_{it} = \frac{\rho_i}{T}\sum_{t=2}^{T}\sum_{a=1}^{t-1} \alpha_i (1 - \alpha_i)^{t-1-a} r_a.$$

This can be calculated as

$$
\begin{aligned}
\mu(\boldsymbol{V}_i) &= \frac{\rho_i}{T}\sum_{t=2}^{T}\left[\alpha_i r_{t-1} + \alpha_i(1-\alpha_i)r_{t-2} + \alpha_i(1-\alpha_i)^2 r_{t-3} + ... + \alpha_i(1-\alpha_i)^{t-2}r_1\right] \\
&= \frac{\rho_i}{T}\begin{pmatrix}
\alpha_i r_{T-1} & +\alpha_i(1-\alpha_i)r_{T-2} & +\alpha_i(1-\alpha_i)^2 r_{T-3} + ... \\
+\,\alpha_i r_{T-2} & +\alpha_i(1-\alpha_i)r_{T-3} & +\alpha_i(1-\alpha_i)^2 r_{T-4} + ... \\
+\,\alpha_i r_{T-3} & +\alpha_i(1-\alpha_i)r_{T-4} & +\alpha_i(1-\alpha_i)^2 r_{T-5} + ... \\
+... & & \\
+\,\alpha_i r_2 & +\alpha_i(1-\alpha_i)r_1 & \\
+\,\alpha_i r_1 & &
\end{pmatrix} \\
&= \frac{\rho_i}{T}\sum_{n=1}^{T-1} r_{T-n}\sum_{c=0}^{n-1}\alpha_i(1-\alpha_i)^c \\
&= \frac{\rho_i}{T}\sum_{n=1}^{T-1} r_{T-n}(1-(1-\alpha_i)^n) \\
&= \frac{\rho_i}{T}\sum_{n=1}^{T-1} r_{T-n} - \frac{\rho_i}{T}\sum_{n=1}^{T-1} r_{T-n}(1-\alpha_i)^n.
\end{aligned}
$$

Here, we have used the formula of the sum of a geometric series

$$\sum_{c=0}^{n-1}\alpha_i(1-\alpha_i)^c = 1-(1-\alpha_i)^n.$$

When T is large and α_i is not extremely close to zero, the second term can be neglected and we obtain the approximation:

$$\mu(\boldsymbol{V}_i) \approx \rho_i\mu(\boldsymbol{r}) \approx \rho_i p_r.$$

From this, the mean of RPE is approximated by

$$\mu(\boldsymbol{\delta}_i) = \rho_i\mu(\boldsymbol{r}) - \mu(\boldsymbol{V}_i) \approx 0.$$

We next calculate the dot product or the square sum of values, $\boldsymbol{V}_i'\boldsymbol{V}_j$:

$$\begin{aligned}
\boldsymbol{V}_i'\boldsymbol{V}_j &= \rho_i\rho_j \sum_{t=2}^{T}\sum_{a=1}^{t-1}\alpha_i(1-\alpha_i)^{t-1-a}r_a\sum_{b=1}^{t-1}\alpha_j(1-\alpha_j)^{t-1-b}r_b \\
&= \rho_i\rho_j\alpha_i\alpha_j\sum_{t=2}^{T}\begin{pmatrix}
r_{t-1}^2 & +(1-\alpha_i)r_{t-2}r_{t-1} & +(1-\alpha_i)^2 r_{t-3}r_{t-1}+... \\
+(1-\alpha_j)r_{t-1}r_{t-2} & +(1-\alpha_i)(1-\alpha_j)r_{t-2}^2 & +(1-\alpha_i)^2(1-\alpha_j)r_{t-3}r_{t-2}+... \\
+(1-\alpha_j)^2 r_{t-1}r_{t-3} & +(1-\alpha_i)(1-\alpha_j)^2 r_{t-2}r_{t-3} & +(1-\alpha_i)^2(1-\alpha_j)^2 r_{t-3}^2+... \\
+... & & \\
+(1-\alpha_j)^{t-2}r_{t-1}r_1 & +(1-\alpha_i)(1-\alpha_j)^{t-2}r_{t-2}r_1 & +(1-\alpha_i)^2(1-\alpha_j)^{t-2}r_{t-3}r_1^2+...
\end{pmatrix} \\
&= \rho_i\rho_j\alpha_i\alpha_j\sum_{t=2}^{T}\Bigg[\sum_{d=0}^{t-2}(1-\alpha_i)^d(1-\alpha_j)^d r_{t-1-d}^2 \\
&\quad + \sum_{\Delta=1}^{t-2}\sum_{d=0}^{t-\Delta-1}(1-\alpha_i)^d(1-\alpha_j)^d\left((1-\alpha_i)^\Delta+(1-\alpha_j)^\Delta\right)r_{t-1-d}r_{t-1-d-\Delta}\Bigg] \\
&= \rho_i\rho_j\alpha_i\alpha_j\Bigg[\sum_{n=0}^{T-2}r_{T-1-n}^2\sum_{m=0}^{n}(1-\alpha_i)^m(1-\alpha_j)^m \\
&\quad + \sum_{\Delta=1}^{T-2}\left((1-\alpha_i)^\Delta+(1-\alpha_j)^\Delta\right)\sum_{n=0}^{T-\Delta-1}r_{T-1-n}r_{T-1-n-\Delta}\sum_{m=0}^{n}(1-\alpha_i)^m(1-\alpha_j)^m\Bigg]
\end{aligned}$$

We approximate each part as

$$\begin{aligned}
\sum_{n=0}^{T-2}r_{T-1-n}^2\sum_{m=0}^{n}(1-\alpha_i)^m(1-\alpha_j)^m &= \sum_{n=0}^{T-2}r_{T-1-n}^2\frac{1-(1-\alpha_i)^n(1-\alpha_j)^n}{1-(1-\alpha_i)(1-\alpha_j)} \\
&= \sum_{n=0}^{T-2}r_{T-1-n}^2\frac{1-(1-\alpha_i)^n(1-\alpha_j)^n}{\alpha_i+\alpha_j-\alpha_i\alpha_j} \\
&\approx \frac{T\mu(\boldsymbol{r}^2)}{\alpha_i+\alpha_j-\alpha_i\alpha_j}
\end{aligned}$$

and

$$\begin{aligned}
&\sum_{n=0}^{T-\Delta-2}r_{T-1-n}r_{T-1-n-\Delta}\sum_{m=0}^{n}(1-\alpha_i)^m(1-\alpha_j)^m \\
&= \sum_{n=0}^{T-\Delta-2}r_{T-1-n}r_{T-1-n-\Delta}\frac{1-(1-\alpha_i)^n(1-\alpha_j)^n}{\alpha_i+\alpha_j-\alpha_i\alpha_j} \\
&\approx \frac{1}{\alpha_i+\alpha_j-\alpha_i\alpha_j}\sum_{n=0}^{T-\Delta-2}r_{T-1-n}r_{T-1-n-\Delta} \\
&= \frac{T-\Delta}{\alpha_i+\alpha_j-\alpha_i\alpha_j}R_\Delta(\boldsymbol{r}).
\end{aligned}$$

Here, we have defined

$$R_\Delta(\boldsymbol{r}) = \frac{1}{T-\Delta}\sum_{a=0}^{T-\Delta} r_a r_{a+\Delta}.$$

Taken above together,

$$\boldsymbol{V}_i'\boldsymbol{V}_j \approx \frac{\rho_i\rho_j\alpha_i\alpha_j}{\alpha_i+\alpha_j-\alpha_i\alpha_j}\left(T\mu(\boldsymbol{r}^2) + \sum_{\Delta=1}^{T-2}\left((1-\alpha_i)^\Delta + (1-\alpha_j)^\Delta\right)(T-\Delta)R_\Delta(\boldsymbol{r})\right).$$

When the reward probability is fixed to p_r, and thus $\mu(\boldsymbol{r}^2) \approx p_r$, $R_\Delta(\boldsymbol{r}) \approx p_r^2$ for $\Delta \neq 0$, we get

$$\boldsymbol{V}_i'\boldsymbol{V}_j \approx \frac{\rho_i\rho_j\alpha_i\alpha_j}{\alpha_i+\alpha_j-\alpha_i\alpha_j}\left(Tp_r + p_r^2\sum_{\Delta=1}^{T-1}\left((1-\alpha_i)^\Delta + (1-\alpha_j)^\Delta\right)(T-\Delta)\right).$$

Using the approximation

$$\sum_{\Delta=1}^{T-1}(1-\alpha_i)^\Delta \approx \frac{1-\alpha_i}{\alpha_i},$$

we arrive at

$$\boldsymbol{V}_i'\boldsymbol{V}_j \approx T\cdot\frac{\rho_i\rho_j\alpha_i\alpha_j}{\alpha_i+\alpha_j-\alpha_i\alpha_j}\left[p_r + p_r^2\left(\frac{1-\alpha_i}{\alpha_i} + \frac{1-\alpha_j}{\alpha_j}\right)\right].$$

When $i = j$ (the squared sum),

$$\boldsymbol{V}_i'\boldsymbol{V}_i \approx T\cdot\frac{\rho_i^2\alpha_i}{2-\alpha_i}\left(p_r + 2p_r^2\frac{1-\alpha_i}{\alpha_i}\right).$$

Next, we evaluate the square sum of RPE,

$$\begin{aligned}\boldsymbol{\delta}_i'\boldsymbol{\delta}_j &= (\rho_i\boldsymbol{r} - \boldsymbol{V}_i)'(\rho_j\boldsymbol{r} - \boldsymbol{V}_j)\\ &= \rho_i\rho_j\boldsymbol{r}'\boldsymbol{r} - \rho_j\boldsymbol{r}'\boldsymbol{V}_j - \rho_i\boldsymbol{r}'\boldsymbol{V}_j + \boldsymbol{V}_i'\boldsymbol{V}_j.\end{aligned}$$

To compute this, we evaluated the inner product of reward and value,

$$\begin{aligned}\boldsymbol{r}'\boldsymbol{V}_i &= \rho_i\sum_{t=2}^{T} r_t\sum_{a=1}^{t-1}\alpha_i(1-\alpha_i)^{t-1-a}r_a\\ &= \rho_i\sum_{\Delta=2}^{T-1}\alpha_i(1-\alpha_i)^{\Delta-1}\sum_{t=1}^{T-\Delta} r_t r_{t+\Delta}\\ &= \rho_i\sum_{\Delta=2}^{T-1}\alpha_i(1-\alpha_i)^{\Delta-1}(T-\Delta)R_\Delta(\boldsymbol{r}).\end{aligned}$$

Note that for $t = 1$, $r_1V_{i1} = 0$.

When reward probability is fixed to p_r,

$$\boldsymbol{r}'\boldsymbol{V}_i \approx T\rho_i p_r^2.$$

From above, when reward probability is fixed to p_r,

$$\begin{aligned}\boldsymbol{\delta}_i'\boldsymbol{\delta}_j &= \rho_i\rho_j\left[\boldsymbol{r}'\boldsymbol{r} - \boldsymbol{r}'\boldsymbol{V}_i - \boldsymbol{r}'\boldsymbol{V}_j + \boldsymbol{V}_i'\boldsymbol{V}_j\right] \\ &= \frac{\rho_i\rho_j(\alpha_i+\alpha_j)}{\alpha_i+\alpha_j-\alpha_i\alpha_j} p_r(1-p_r),\end{aligned}$$

and

$$\boldsymbol{\delta}_i'\boldsymbol{\delta}_i = T\frac{2\rho_i^2}{2-\alpha_i} p_r(1-p_r).$$

Finally, we evaluate the dot product of RPE and reward, $\boldsymbol{\delta}_i, \boldsymbol{r}$:

$$\begin{aligned}\boldsymbol{\delta}_i'\boldsymbol{r} &= (\rho_i\boldsymbol{r} - \boldsymbol{V}_i)'\boldsymbol{r} \\ &= \rho_i\boldsymbol{r}'\boldsymbol{r} - \boldsymbol{V}_i'\boldsymbol{r} \\ &= \rho_i\boldsymbol{r}'\boldsymbol{r} - \sum_{\Delta=1}^{T-1}\alpha_i(1-\alpha_i)^{\Delta-1}(T-\Delta)R_\Delta(\boldsymbol{r}).\end{aligned}$$

For the fixed reward probability p_r,

$$\begin{aligned}\boldsymbol{\delta}_i'\boldsymbol{r} &= T\rho_i p_r - \rho_i p_r^2\sum_{\Delta=1}^{T-1}\alpha_i(1-\alpha_i)^{\Delta-1}(T-\Delta) \\ &\approx T\rho_i p_r(1-p_r).\end{aligned}$$

2 Derivation of estimates for GLMs

To obtain the component-wise expression for estimates of regression coefficient in Eq 13 in the main text, we provide results of matrix calculations.

$$X'X = \begin{bmatrix} x_{11} & x_{21} & \dots & x_{T1} \\ x_{12} & x_{22} & \dots & x_{T2} \end{bmatrix} \begin{bmatrix} x_{11} & x_{12} \\ x_{21} & x_{22} \\ \vdots & \vdots \\ x_{T1} & x_{T2} \end{bmatrix} = \begin{bmatrix} S(\boldsymbol{x}_1, \boldsymbol{x}_1) & S(\boldsymbol{x}_1, \boldsymbol{x}_2) \\ S(\boldsymbol{x}_1, \boldsymbol{x}_2) & S(\boldsymbol{x}_2, \boldsymbol{x}_2) \end{bmatrix},$$

$$(X'X)^{-1} = \frac{1}{S(\boldsymbol{x}_1, \boldsymbol{x}_1)S(\boldsymbol{x}_2, \boldsymbol{x}_2) - S(\boldsymbol{x}_1, \boldsymbol{x}_2)^2} \begin{bmatrix} S(\boldsymbol{x}_2, \boldsymbol{x}_2) & -S(\boldsymbol{x}_1, \boldsymbol{x}_2) \\ -S(\boldsymbol{x}_1, \boldsymbol{x}_2) & S(\boldsymbol{x}_1, \boldsymbol{x}_1) \end{bmatrix},$$

$$X'Y = \begin{bmatrix} x_{11} & x_{21} & \dots & x_{T1} \\ x_{12} & x_{22} & \dots & x_{T2} \end{bmatrix} \begin{bmatrix} y_1 \\ y_2 \\ \vdots \\ y_T \end{bmatrix} = \begin{bmatrix} S(\boldsymbol{x}_1, Y) \\ S(\boldsymbol{x}_2, Y) \end{bmatrix}.$$

From these, the estimate of regression coefficient, β, is expressed as

$$\begin{aligned} \begin{bmatrix} \hat{\beta}_1 \\ \hat{\beta}_2 \end{bmatrix} &= (X'X)^{-1}X'Y = \frac{1}{S(\boldsymbol{x}_1, \boldsymbol{x}_1)S(\boldsymbol{x}_2, \boldsymbol{x}_2) - S(\boldsymbol{x}_1, \boldsymbol{x}_2)^2} \begin{bmatrix} S(\boldsymbol{x}_2, \boldsymbol{x}_2) & -S(\boldsymbol{x}_1, \boldsymbol{x}_2) \\ -S(\boldsymbol{x}_1, \boldsymbol{x}_2) & S(\boldsymbol{x}_1, \boldsymbol{x}_1) \end{bmatrix} \begin{bmatrix} S(\boldsymbol{x}_1, Y) \\ S(\boldsymbol{x}_2, Y) \end{bmatrix} \\ &= \frac{1}{S(\boldsymbol{x}_1, \boldsymbol{x}_1)S(\boldsymbol{x}_2, \boldsymbol{x}_2) - S(\boldsymbol{x}_1, \boldsymbol{x}_2)^2} \begin{bmatrix} S(\boldsymbol{x}_2, \boldsymbol{x}_2)S(\boldsymbol{x}_1, Y) - S(\boldsymbol{x}_1, \boldsymbol{x}_2)S(\boldsymbol{x}_2, Y) \\ -S(\boldsymbol{x}_1, \boldsymbol{x}_2)S(\boldsymbol{x}_1, Y) + S(\boldsymbol{x}_1, \boldsymbol{x}_1)S(\boldsymbol{x}_2, Y) \end{bmatrix}, \end{aligned}$$

which leads to Eq 16 in the main text.

3 Statistics for regression coefficients

Next, we provide component-wise expression of the mean and variance of the estimates of regression coefficients, given as Eq 19 in the main text.

First, we calculate the expected value of $\hat{\boldsymbol{\beta}}$: From

$$
\begin{aligned}
X'X^*\boldsymbol{\beta} &= \begin{bmatrix} x_{11} & x_{21} & \dots & x_{T1} \\ x_{12} & x_{22} & \dots & x_{T2} \end{bmatrix} \begin{bmatrix} x^*_{11} & x^*_{12} \\ x^*_{21} & x^*_{22} \\ \vdots & \vdots \\ x^*_{T1} & x^*_{T2} \end{bmatrix} \begin{bmatrix} \beta_1 \\ \beta_2 \end{bmatrix} \\
&= \begin{bmatrix} S(x_1, x^*_1) & S(x_1, x^*_2) \\ S(x^*_1, x_2) & S(x_2, x^*_2) \end{bmatrix} \begin{bmatrix} \beta_1 \\ \beta_2 \end{bmatrix},
\end{aligned}
$$

we obtain

$$
\begin{aligned}
\mathrm{E}[\hat{\boldsymbol{\beta}}] &= \frac{1}{S(x_1, x_1)S(x_2, x_2) - S(x_1, x_2)^2} \\
&\times \begin{bmatrix} S(x_2, x_2) & -S(x_1, x_2) \\ -S(x_1, x_2) & S(x_1, x_1) \end{bmatrix} \begin{bmatrix} S(x_1, x^*_1) & S(x_1, x^*_2) \\ S(x^*_1, x_2) & S(x_2, x^*_2) \end{bmatrix} \begin{bmatrix} \beta_1 \\ \beta_2 \end{bmatrix} \\
&= \frac{1}{S(x_1, x_1)S(x_2, x_2) - S(x_1, x_2)^2} \\
&\times \begin{bmatrix} S(x_2, x_2)S(x_1, x^*_1) - S(x_1, x_2)S(x^*_1, x_2) & S(x_2, x_2)S(x_1, x^*_2) - S(x_1, x_2)S(x^*_2, x_2) \\ -S(x_1, x_2)S(x_1, x^*_1) + S(x_1, x_1)S(x^*_1, x_2) & -S(x_1, x_2)S(x_1, x^*_2) + S(x_1, x_1)S(x_2, x^*_2) \end{bmatrix} \begin{bmatrix} \beta_1 \\ \beta_2 \end{bmatrix}.
\end{aligned}
$$

From this, Eqs 20 and 21 are obtained.

References

1. Wilson RC, Niv Y. Is Model Fitting Necessary for Model-Based fMRI? PLoS Computational Biology. 2015;11(6):e1004237.
