# Supplementary material for: Revisiting the importance of model fitting for model-based fMRI: It does matter in computational psychiatry
Source: PLoS Comput Biol. 2021 Feb 9;17(2):e1008738. doi: 10.1371/journal.pcbi.1008738 (PMC7899379; doi:10.1371/journal.pcbi.1008738)
Supplement: S2 Text — (PDF) [file pcbi.1008738.s002.pdf]

Supplementary Material (S2 Text)
Revisiting the importance of model fitting for model-based fMRI: It does matter in computational psychiatry

Kentaro Katahira[1], Asako Toyama[1]

1 Department of Psychological and Cognitive Sciences, Nagoya University, Nagoya, Japan

1 Effects of reward sensitivity in Rescorla-Wagner model

Here, we examined the effects of misfit of reward sensitivity, ρ, in a similar way to that for the effects of misfit of learning rate, α, in the main text.

Fig 1A and Fig 1B show the time courses of the latent variables obtained by simulations for three different values of ρ while the learning rate is set to $\alpha = 0.4$. Other settings are the same as those in Fig 1 in the main text. It is evident that ρ modulates the scale of the latent variables without influencing the shape of the trajectory. The greater ρ is, the greater the variances (magnitudes) of V and δ are.

Fig 1C and 1D show a correlation between hypothetical BOLD signal, y_t and the value signal V_t and RPE signal δ_t, respectively. To generate y_t, we set the true parameters as $\alpha = 0.4$ and $\rho = 1.0$ and true regression coefficients as $\beta_V = 1$ (for Fig 1C), and $\beta_\delta = 1$ (for Fig 1D). While the estimates of the regression coefficients were scaled by the magnitude of ρ, their correlation coefficient is not influenced by changing ρ.

Next, we consider a simulation where activities to RPE are compared between individuals with depression (Group 1, $\rho = 0.9$) and those of healthy controls (Group 2, $\rho = 1.1$) . This setting of reward sensitivity is in accordance with studies reporting that reward sensitivity is smaller in individuals with depression [1, 2]. We assumed that the common parameter, $\hat{\rho} = 1.0$, is used to both groups to derive the estimate of RPE signal, $\hat{\delta}$.

Fig 2A shows the result of GLM1. The estimate of the regression coefficient for RPE, $\hat{\beta}_\delta$, significantly differed between two groups ($t_{38} = -7.41, p < 0.001$, unpaired t-test). In GLM2 (Fig 2B), the both regression coefficients for reward and negative value differed between two groups ($t_{38} = -6.54, p < 0.001$, $t_{38} = -3.54, p = 0.001$, respectively).

The analytical values of the expected value and s.d. of regression coefficients, which are provided in Materials and methods, are plotted in Fig 3 as a function of fit reward sensitivity, $\hat{\rho}$. The influence of reward

sensitivity on the expected value of $\hat{\beta}_\delta$, are identical both for GLM1 and GLM2, while its variance is smaller for GLM1. This is why GLM1 attained highly significant results compared to GLM2. The effect size for a one sample t-test is not influenced by fit ρ (Fig 3E and 3F). The larger the true reward sensitivity, the larger the effect size.

References

1. Kunisato Y, Okamoto Y, Ueda K, Onoda K, Okada G, Yoshimura S, et al. Effects of depression on reward-based decision making and variability of action in probabilistic learning. Journal of Behavior Therapy and Experimental Psychiatry. 2012;43(4):1088–94. doi:10.1016/j.jbtep.2012.05.007.

2. Huys QJ, Pizzagalli DA, Bogdan R, Dayan P. Mapping anhedonia onto reinforcement learning: a behavioural meta-analysis. Biol Mood Anxiety Disord. 2013;3(1):12.

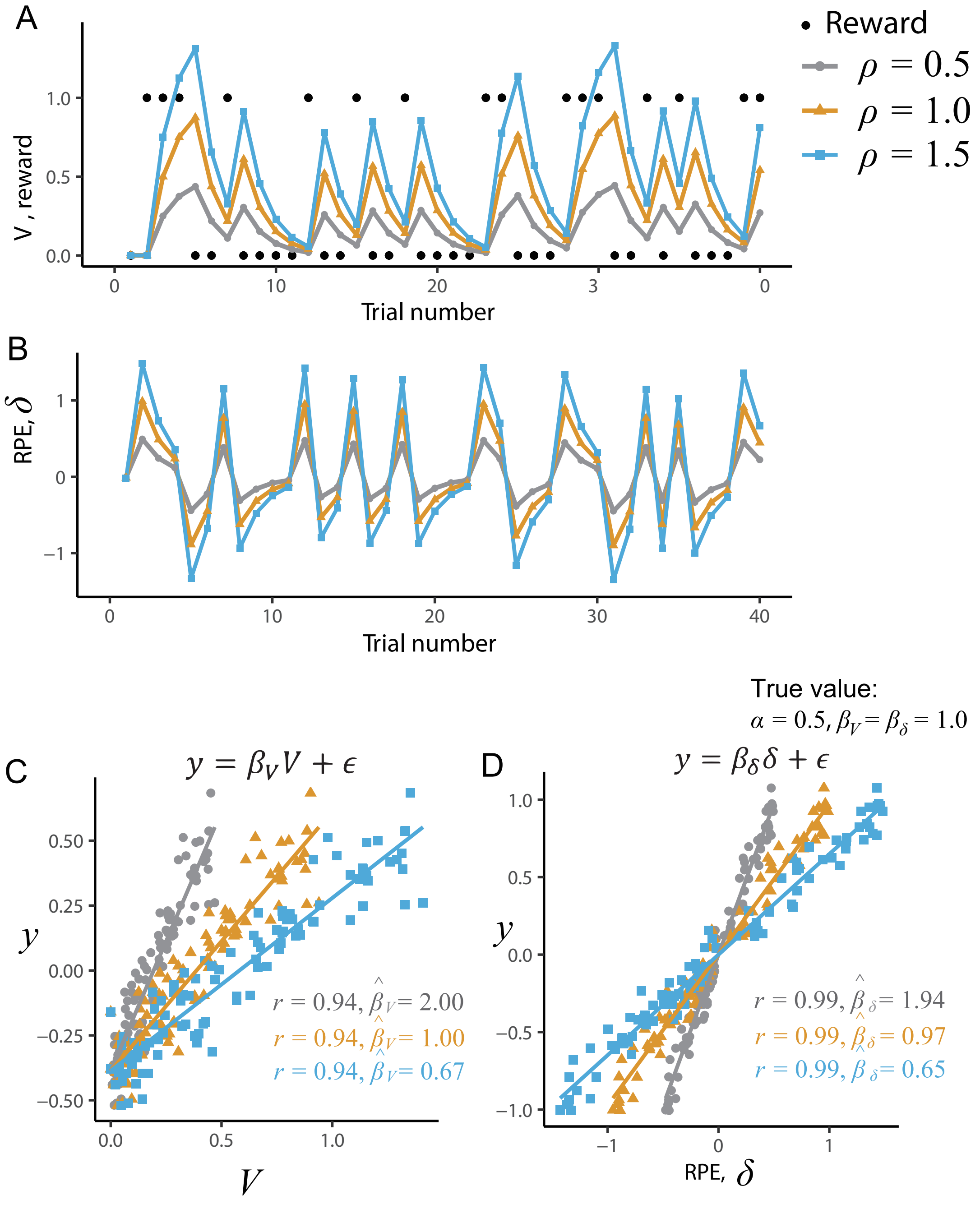

Fig 1. Examples of the simulation of Rescorla-Wagner model with varying reward sensitivity, ρ. (A, B) Time courses of the value, V, and RPE, δ, respectively. (C, D) The effects of learning rate on the correlation between the value (C) / RPE (D) and hypothetical neural signals, which were generated by the linear regression model.

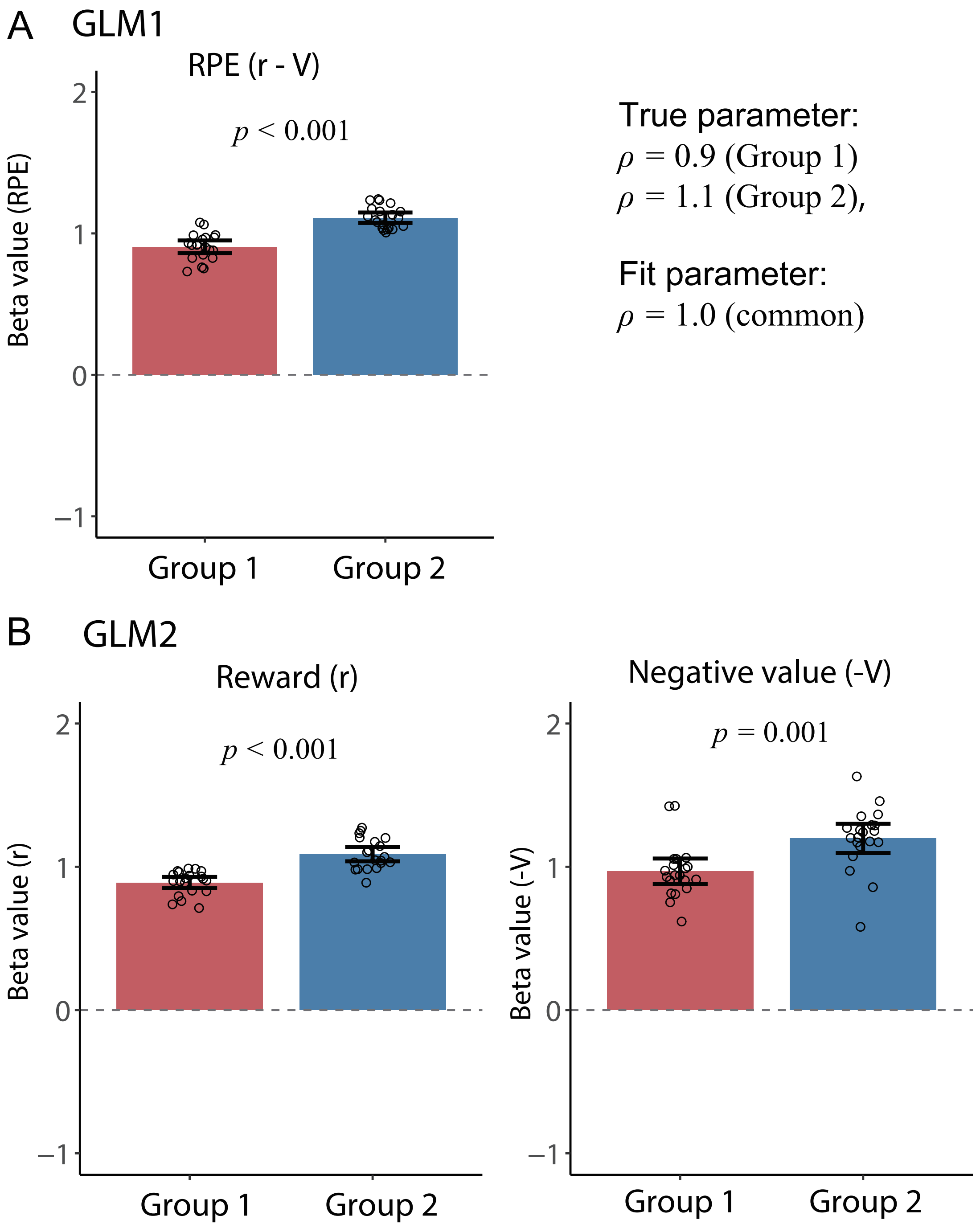

Fig 2. Simulations of group comparison of regression coefficients for RPE. The GLMs are fit to the hypothetical neural signals that are generated based on RW model with different reward sensitivities between groups. (A) GLM1 (RPE is a sole regressor). (B) GLM2 (RPE and reward are regressors). The error bars indicate 95% confidence interval.

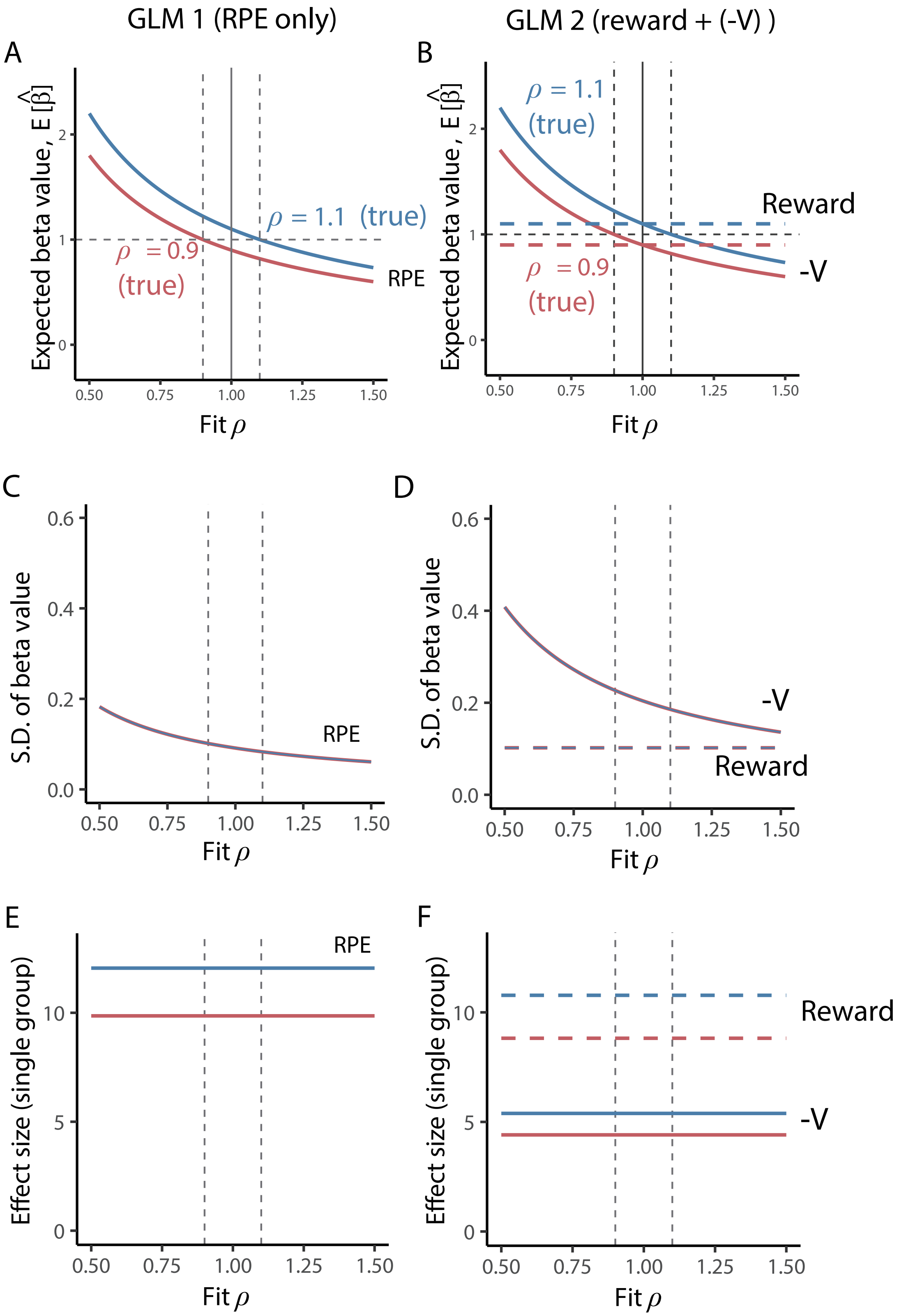

Fig 3. Analytical results for group comparison of regression coefficients in Rescorla-Wagner model. The convention is the same as that in Fig 3 of the main text except that red lines indicate true reward sensitivity $\rho = 0.9$ and blue lines indicate $\rho = 1.1$.
