# Supplementary material for: Revisiting the importance of model fitting for model-based fMRI: It does matter in computational psychiatry
Source: PLoS Comput Biol. 2021 Feb 9;17(2):e1008738. doi: 10.1371/journal.pcbi.1008738 (PMC7899379; doi:10.1371/journal.pcbi.1008738)
Supplement: S3 Text — (PDF) [file pcbi.1008738.s003.pdf]

Supplementary Material (S3 Text)

Revisiting the importance of model fitting for model-based fMRI: It does matter in computational psychiatry

Kentaro Katahira[1], Asako Toyama[1]

[1]Department of Psychological and Cognitive Sciences, Nagoya University, Nagoya, Japan

Effects of ground-truth learning rates

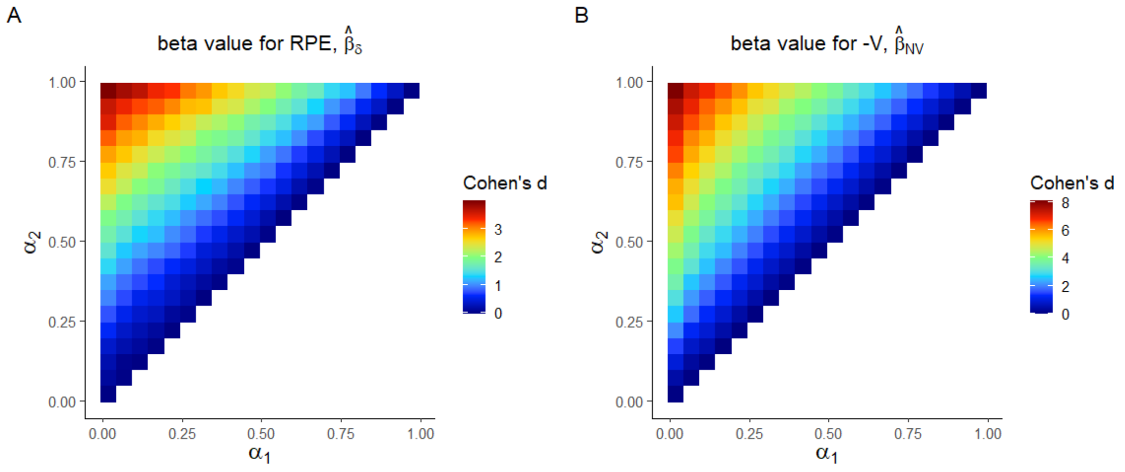

Fig 1. Dependence of effect size on the combinations of the ground-truth learning rates. The heatmap indicates the effect size of between-group difference (Cohen's d), for the beta value for RPE (panel A) and that for negative value (panel B).

In the main text, we set the true learning rates for both groups to 0.2 and 0.4, respectively. Here we examine how the effect size regarding group differences depends on the different combinations of learning rates. Other conditions are identical to the simulation of classical conditioning tasks in the main text. Note that analytical expressions do not hold when learning rate is near 0 or 1 [1]. Thus, here we performed numerical simulations without relying on analytical results.

The simulation is conducted with all possible combinations of ground-truth learning rates for Low-L group (α_1) and High-L group (α_2) in the range [0.01, 0.99] with increments of 0.02. Due to the symmetry between groups (e.g., the effect size when $\alpha_1 = 0.4,$ and $\alpha_2 = 0.2$ should be equal to the negative of the effect size when $\alpha_1 = 0.2,$ and $\alpha_2 = 0.4$), simulations

were conducted only for conditions where $\alpha_1 < \alpha_2$. The fit learning rate commonly used for both groups is set to the mean of true learning rates (i.e., $\hat{\alpha} = (\alpha_1 + \alpha_2)/2$).

The heatmap in Fig 1 shows the effect size averaged over 200 simulations. In both beta values for RPE (panel A) and negative value (panel B), the effect size of group differences mainly depends on relative group differences in learning rates, rather than in absolute values (e.g., the effect size for $\alpha_1 = 0.1,$ and $\alpha_2 = 0.3$ yields a similar effect size for $\alpha_1 = 0.2,$ and $\alpha_2 = 0.4$). This allows us to focus on the relative difference in learning rate, rather than their absolute values.

References

1. Wilson RC, Niv Y. Is Model Fitting Necessary for Model-Based fMRI? PLoS Computational Biology. 2015;11(6):e1004237.
