# Supplementary material for: Revisiting the importance of model fitting for model-based fMRI: It does matter in computational psychiatry
Source: PLoS Comput Biol. 2021 Feb 9;17(2):e1008738. doi: 10.1371/journal.pcbi.1008738 (PMC7899379; doi:10.1371/journal.pcbi.1008738)
Supplement: S4 Text — (PDF) [file pcbi.1008738.s004.pdf]

Supplementary Material (S4 Text)

Revisiting the importance of model fitting for model-based fMRI: It does matter in computational psychiatry

Kentaro Katahira[1], Asako Toyama[1]
1 Department of Psychological and Cognitive Sciences, Nagoya University, Nagoya, Japan

Effects of reward contingencies

Here we consider the influences of reward contingencies in a classical conditioning task on the effect size of group differences in beta values (regression coefficients). Specifically, as reward contingencies, we consider drifting reward mean (Gaussian random walk) with gaussian reward, drifting reward probability with binary reward, and reward probability switching.

Drifting reward mean with gaussian reward

First, we consider the case where rewards are drawn from a Gaussian distribution whose mean, m_t, changes following a random walk:

$$r_t \sim N(m_t, \sigma_n^2), \quad (1)$$

$$m_{\mathrm{t+1}} = \gamma m_t + \epsilon_t, \ (2)$$

where $\epsilon_t$obeys a Gaussian distribution with zero mean and drift variance σ_d^2. $\gamma\ (< 1)$ is a decay parameter. A larger γ and larger σ_d^2 leads to a large variance of reward magnitude and a greater autocorrelation of reward sequences (see Eq 23 in [1]).

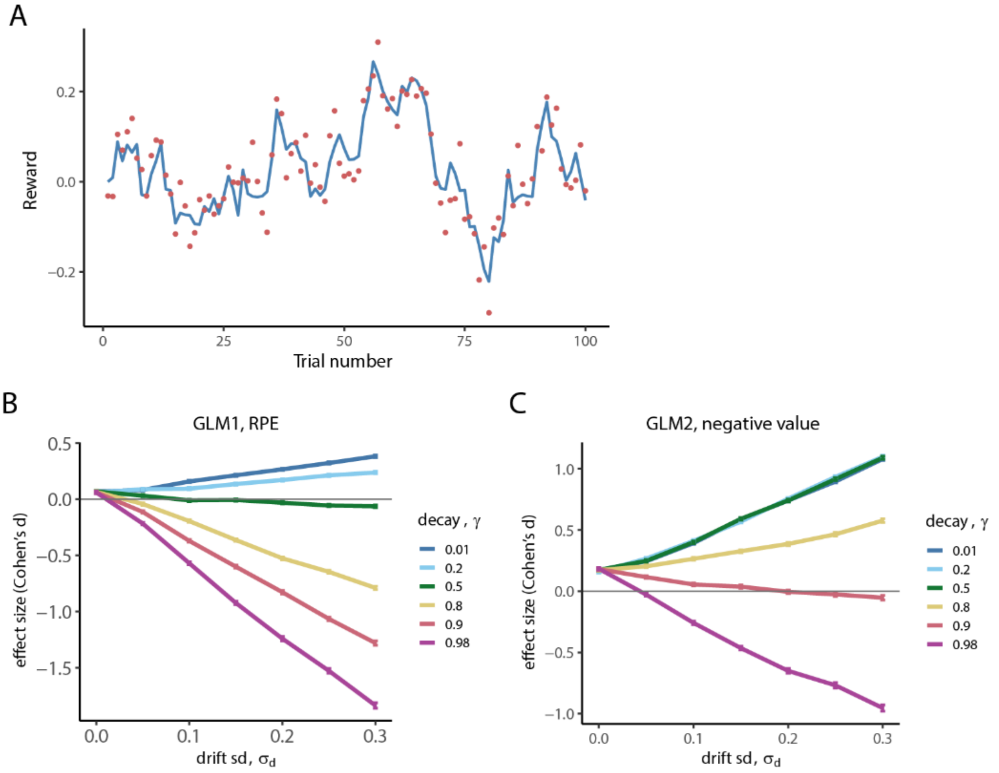

Fig 1. Effects of drifting continuous reward. (A) Typical reward sequence of drifting mean reward condition, where the mean reward obeys drifting Gaussian random walk with $\gamma = 0.98$ $\sigma_d^2 = 0.5,$ and $\sigma_n^2 = 0.05$. The blue line indicates the mean reward value, m_t, while the red dots represent actual rewards, r_t. (B) The effect size of group differences (Cohen's d), where positive value indicates that high learning rate (High-L) group, and whose learning rate is 0.4, has larger beta-values than the Low-L group, whose learning rate is 0.2. Cohen's d is plotted for different drift and decay rates. The means are taken for 200 simulations for each condition. Although negligible, error bars represent the standard error of the mean (s.e.m.),.

We performed simulations by varying the drift variance (σ_d^2) and decay rate (γ). Other settings were the same as the classical conditioning paradigm in the main text. Fig 1B and C show the effect size of group differences. As the drift variance (σ_d^2) increases, the magnitude of the effect size of group differences in beta-values for RPE (panel B) and negative value (panel C) increases. As γ was close to one (weak decay), the effect size decreases and even became negative as the drift variance increases. The negative effect size indicates that the Low-L group, where the true learning rate is smaller than the High-L group, show larger beta-values compared to the High-L group. In what follows, we consider the mechanism underlying this result (i.e., opposite effect size).

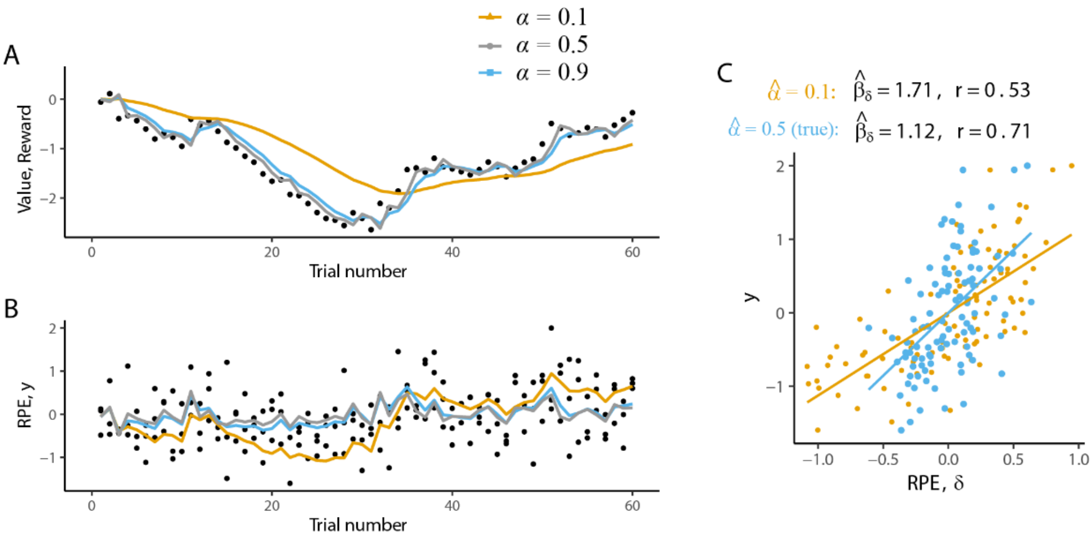

Fig 2. Example model behavior for illustrating the mechanism by which autocorrelation leads to negative effect size. (A,B) How learning rates changes the development of value (A) and RPEs (B). (C) Correlation between RPE signal and generated neural signal, y. Here we generated neural signals with the ground-truth learning rate being $\alpha = 0.5$. Results of two cases of fit learning rate,$\hat{\alpha}$ = 0.1 (orange), and $\hat{\alpha}$ = 0.5 (blue) are plotted.

Fig 2A and 2B show an example sequence of reward and value when the degree of drift is strong ($\gamma = 0.98$, $\sigma_d^2 = 2.0$, and $\sigma_n^2 = 0.5$). To clarify the effect, we considered the overly different learning rates, $\alpha = 0.1, 0.5$ and 0.9. When the learning rate is small ($\alpha = 0.1$, orange line), the model cannot track the dynamics of the drifting mean, in contrast to the case where larger learning rates are used ($\alpha = 0.5, 0.9$, blue and gray lines, respectively). Thus, smaller learning rates produce larger variance of RPE (Fig 2B, 2C). Note that the variance of RPE depends on the combination of reward contingency and learning rates: remember the case with the fixed reward probability, where a larger learning rate causes a larger variance of RPE (as shown in Fig 1 in the main text). For the beta-values for RPE, when the true learning rate is small (e.g., $\alpha = 0.1$, orange line), while the degree of the drift of reward is large (Fig 2A), using a larger learning rate (e.g., $\alpha = 0.5$, blue line) as a fit parameter, inflated the beta-value of RPE (orange line, $\hat{\beta}_\delta = 1.71$) due to a smaller variance of RPE (regressor) compared to when the true learning rate is used (blue line, $\hat{\beta}_\delta = 1.12$) （Fig 2B); even the correlation between the regressor ($\hat{\delta}$) and the neural variable (y) was smaller ($r = 0.53$) than when the true learning rate is used ($r = 0.71$), the beta-value was larger compared to when the true learning rate (=0.1) was used）. In consequence, the group with small learning rates have larger beta-value for RPE, compared to that with larger learning rates, if one uses a common learning rate for both groups to obtain RPE signals. This leads to the

negative effect size.

The mechanism of the increase of the magnitude of effect sizes (in both direction) due to increase in drift variance (σ_d^2) is rather simple. Larger drift noise induces a larger magnitude of reward, while we fixed the variance of fMRI noise (σ_ϵ^2) constant. This renders the magnitude of the value and RPE signals large, and thus signal-to-noise ratio gets larger with increasing drift noise, leading to a higher effect size of the difference between groups.

Drifting reward probability (binary reward)

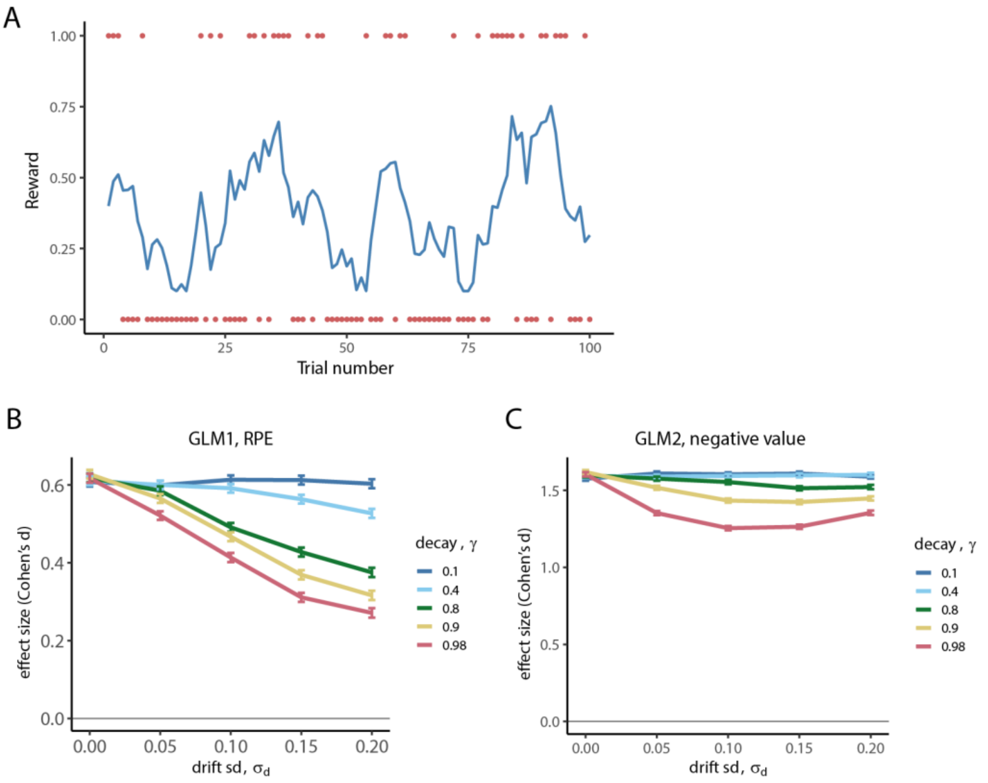

Fig 3. Effects of drifting reward probability in binary reward case. A. Typical reward sequence of drifting reward probability, where the probability reward follows a drifting Gaussian random walk with $\gamma = 0.98,\ \sigma_d^2 = 0.1$. The blue line indicates the reward probability while the red dots represent actual rewards (0 = absence of reward, 1 = presence of reward). B. The effect size of group differences in beta values for RPE (B) and those for negative values (C). The convention is same as in Fig 1.

Next, we consider the case where reward is binary (0 or 1) as in the example of the main text, whereas the reward probability obeys the Gaussian random walk as in Eq. 2, but the reward probability is restricted to the range [0.1, 0.9]: when the random walk deviated from

this range, the value was replaced with the nearest one (0.1 or 0.9). An example of reward sequence generated from this process is shown in Fig 3A, while Fig 3B and 3C show the results of simulations with varying drift variance (σ_d^2) and decay rate (γ). The larger the drift variance (thus larger the autocorrelation) is, the weaker the effect size. Also, larger decay rates (γ) make this decline more pronounced. However, this effect is small compared to continuous (Gaussian) reward cases. This is because the large degree of stochasticity in binary rewards precludes the autocorrelation of reward to become large enough such that the effect is reverted as in the Gaussian reward case.

Switching reward probability (binary reward)

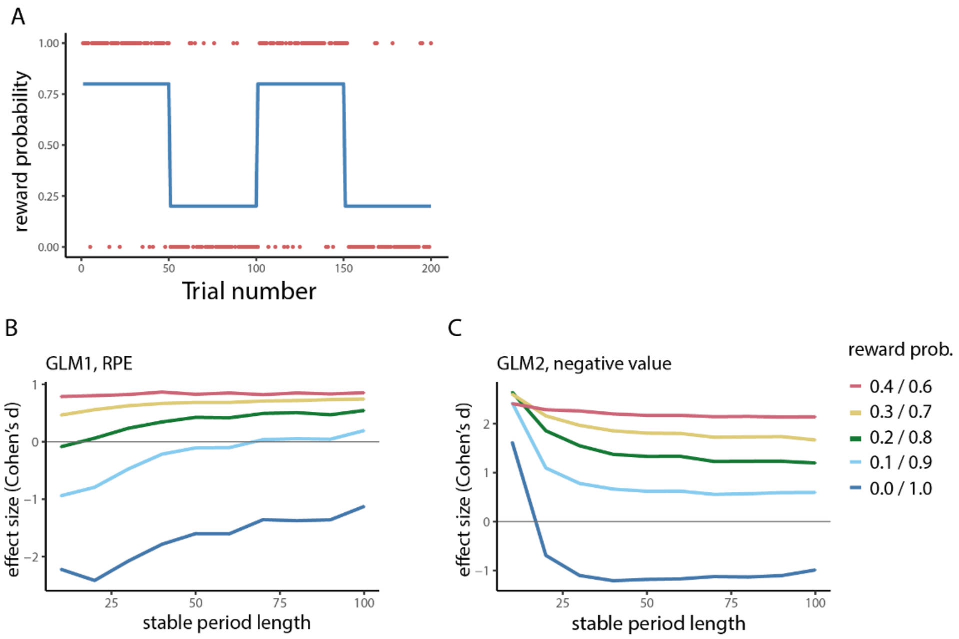

Fig 3. Effects of reward probability switching. A. Typical reward sequence of switching reward probability with stable trial length is 50, and reward probabilities 0.2/0.8. The blue line indicates reward rate while the red dots represent actual rewards. B. The effect size of group difference for RPE (B) and negative value (C) as a function of stable trial length and different reward probabilities. The convention is the same as in Fig 1.

Finally, we consider the situation where the reward contingency is abruptly switched from high and low probabilities. We varied the stable-period length (how many trials elapse after the previous switching or the beginning time for the first switch) and the reward probability set (high/low). The results for different stable-period lengths and different reward probability settings are plotted in Fig 3B and 3C. As the stable-period length is shorter, the beta value for RPE becomes smaller and even becomes negative. This tendency is larger when the contrast in reward probabilities is large (e.g., 0.9/0.1). When the contrast is high, the effect size becomes negative (e.g., 0.9/0.1). These results are explained because of higher

autocorrelation of reward sequences as in the case with drifting reward means. The beta-value of negative values shows an opposite tendency regarding stable-period length: short stable-period length yielded large group differences.

References

1. Wilson RC, Niv Y. Is Model Fitting Necessary for Model-Based fMRI? PLoS Computational Biology. 2015;11(6):e1004237.
