# Supplementary material for: Revisiting the importance of model fitting for model-based fMRI: It does matter in computational psychiatry
Source: PLoS Comput Biol. 2021 Feb 9;17(2):e1008738. doi: 10.1371/journal.pcbi.1008738 (PMC7899379; doi:10.1371/journal.pcbi.1008738)
Supplement: S5 Text — (PDF) [file pcbi.1008738.s005.pdf]

Supplementary Material (S5 Text)
Revisiting the importance of model fitting for model-based fMRI: It does matter in computational psychiatry

Kentaro Katahira[1], Asako Toyama[1]

1 Department of Psychological and Cognitive Sciences, Nagoya University, Nagoya, Japan

1 Effects of normalization of regressors and response variables

In the present study, we have assumed that regressors and response variables (neural signal) are all mean-centered, but we have not normalized them (s.d. were in their original value). In contrast, Wilson & Niv [1] normalized the regressors to have a standard deviation of 1. Here we examine the effect of scaling (normalizing) of regressors and target BOLD signal.

To simplify the problem, here we consider a regression model with a single regressor. We assume that the two groups with different ground-truth regressors $x^{(1)}$, $x^{(2)}$

$$y_t^{(i)} = \beta x_t^{(i)} + \epsilon_t^{(i)},$$
$$\epsilon_t^{(i)} \sim \mathcal{N}(0, \sigma_\epsilon^2) \text{ for } i = 1, 2.$$

We assume that the true regression coefficients and variance of observation noise are common to both groups.

The set of specific values of $x_t^{(i)}$ are denoted by the vector $\boldsymbol{x}^{(i)}$. As in the situation of the main text, the common regressor obtained with fit model parameter is denoted by $\hat{\boldsymbol{x}}$.

From Eq. (22) in the main text, the expected value of the estimate of $\beta^{(i)}$ (for group i) is given as a function of $\boldsymbol{x}^{(i)}$, and $\hat{\boldsymbol{x}}$:

$$\mathrm{E}[\hat{\beta}^{(i)}] = \mathrm{Cor}(\hat{\boldsymbol{x}}, \boldsymbol{x}^{(i)}) \frac{\sqrt{S(\boldsymbol{x}^{(i)}, \boldsymbol{x}^{(i)})}}{\sqrt{S(\hat{\boldsymbol{x}}, \hat{\boldsymbol{x}})}} \beta, \tag{1}$$

where β is the true value for the regression coefficient. From Eq.(29) in the main text, the variance of $\hat{\beta}^{(i)}$ is given by

$$\mathrm{Var}[\hat{\beta}^{(i)}] = \frac{\sigma_\epsilon^2}{\sqrt{S(\hat{\boldsymbol{x}}, \hat{\boldsymbol{x}})}}, \tag{2}$$

The effect size of group difference (Cohen's d) is calculated as

$$d_{12} = \frac{\mathrm{E}[\hat{\beta}^{(1)}] - \mathrm{E}[\hat{\beta}^{(2)}]}{\sqrt{(\mathrm{Var}[\hat{\beta}^{(1)}] + \mathrm{Var}[\hat{\beta}^{(2)}])/2}}, \tag{3}$$

$$= \frac{\mathrm{Cor}(\hat{\boldsymbol{x}}, \boldsymbol{x}^{(1)})\sqrt{S(\boldsymbol{x}^{(1)}, \boldsymbol{x}^{(1)})} - \mathrm{Cor}(\hat{\boldsymbol{x}}, \boldsymbol{x}^{(2)})\sqrt{S(\boldsymbol{x}^{(2)}, \boldsymbol{x}^{(2)})}}{\sigma_\epsilon^2}\beta. \tag{4}$$

Note that the variance of common fit regressors $\hat{\boldsymbol{x}}$ does not influence the effect size. Thus, the effect size is invariant under rescaling (normalization) of fit regressors.

Here we consider the rescaling of $y^{(1)}$ and $y^{(2)}$ as follows:

$$y^{(1)} \rightarrow a^{(1)} y^{(1)},$$
$$y^{(2)} \rightarrow a^{(2)} y^{(2)}$$

After this rescaling, the expected value of beta value becomes

$$\mathrm{E}[\hat{\beta}^{(1)}] = \mathrm{Cor}(\hat{\boldsymbol{x}}, \boldsymbol{x}^{(1)}) \frac{\sqrt{S(\boldsymbol{x}^{(1)}, \boldsymbol{x}^{(1)})}}{\sqrt{S(\hat{\boldsymbol{x}}, \hat{\boldsymbol{x}})}} \beta^{(1)} \cdot a^{(1)}, \tag{5}$$

$$\mathrm{E}[\hat{\beta}^{(2)}] = \mathrm{Cor}(\hat{\boldsymbol{x}}, \boldsymbol{x}^{(2)}) \frac{\sqrt{S(\boldsymbol{x}^{(2)}, \boldsymbol{x}^{(2)})}}{\sqrt{S(\hat{\boldsymbol{x}}, \hat{\boldsymbol{x}})}} \beta^{(2)} \cdot a^{(2)}. \tag{6}$$

We here assume that

$$\mathrm{Var}[x^{(1)}] > \mathrm{Var}[x^{(2)}]$$

and

$$\mathrm{Cor}(\hat{\boldsymbol{x}}, \boldsymbol{x}^{(1)}) \approx \mathrm{Cor}(\hat{\boldsymbol{x}}, \boldsymbol{x}^{(2)}).$$

Then, the effect size becomes

$$d_{12} \propto a^{(1)}\sqrt{S(\boldsymbol{x}^{(1)}, \boldsymbol{x}^{(1)})} - a^{(2)}\sqrt{S(\boldsymbol{x}^{(2)}, \boldsymbol{x}^{(2)})}. \tag{7}$$

Let us consider the effect of normalization of y (so that the variance of y is 1). The variance of $y^{(i)}$ is $\mathrm{var}(y^{(i)}) = \beta^2 \mathrm{var}(x^{(i)}) + \sigma_\epsilon^2$. Thus, the scaling factor $a^{(i)}$ would be

$$a^{(i)} \approx \frac{1}{\sqrt{\beta^2 \mathrm{Var}(x^{(i)}) + \sigma_\epsilon^2}}$$

Therefore, the effect size can be expressed as

$$d_{12} \propto \frac{\sqrt{S(\boldsymbol{x}^{(1)}, \boldsymbol{x}^{(1)})}}{\sqrt{\beta^2 \mathrm{Var}(x^{(1)}) + \sigma_\epsilon^2}} - \frac{\sqrt{S(\boldsymbol{x}^{(2)}, \boldsymbol{x}^{(2)})}}{\sqrt{\beta^2 \mathrm{Var}(x^{(1)}) + \sigma_\epsilon^2}} \tag{8}$$

When σ_ϵ^2 is small enough compared to $\beta^1\text{Var}(x^{(1)})$ and $\beta^2\text{Var}(x^{(2)})$, because $S(\boldsymbol{x}^{(1)}, \boldsymbol{x}^{(1)})/T \approx \text{Var}(x^{(1)})$, $S(\boldsymbol{x}^{(2)}, \boldsymbol{x}^{(2)})/T \approx \text{Var}(x^{(2)})$ for large T, the effect size will be vanished by the normalization of response variable y. However, when σ_ϵ^2 is relatively large and not negligible, the relative impact of the variance in normalization terms, $\text{Var}(x^{(i)})$ will be small. Thus, the difference in variance of true regressors influence the effect size for group differences as when the case without normalization.

Therefore, although the normalization of y can diminish the group difference, the effect of the difference in variance of true x (smaller variance of x leads to smaller estimates for β) remains.

Simulation

To illustrate the impact of normalization of the response variable, y, we performed simulations with or without normalization and with various noise variances. To obtain stable estimates of the effect size, we simulated $n = 50$ hypothetical subjects for each group. We ran 100 simulations for each condition. Other simulation settings were identical with the simulation of the classical conditioning paradigm in the main text.

Fig 1 plots the resulting between-group effect sizes. Overall, as the noise variance increases, the effect size of group differences in beta-values for RPE (orange line) and negative value (blue line) decreases. For RPE (Panel A), as our theoretical consideration predicted, the normalization basically reduces the effect size, but the reduction is evident only when the s.d. of fMRI (observation) noise (σ_ϵ^2) is small. When noise s.d. is 0.5, which is our default setting, the effect of normalization is negligible. For negative values (Panel B), the effect of normalization is small for all ranges of noise variance.

References

1. Wilson RC, Niv Y. Is Model Fitting Necessary for Model-Based fMRI? PLoS Computational Biology. 2015;11(6):e1004237.

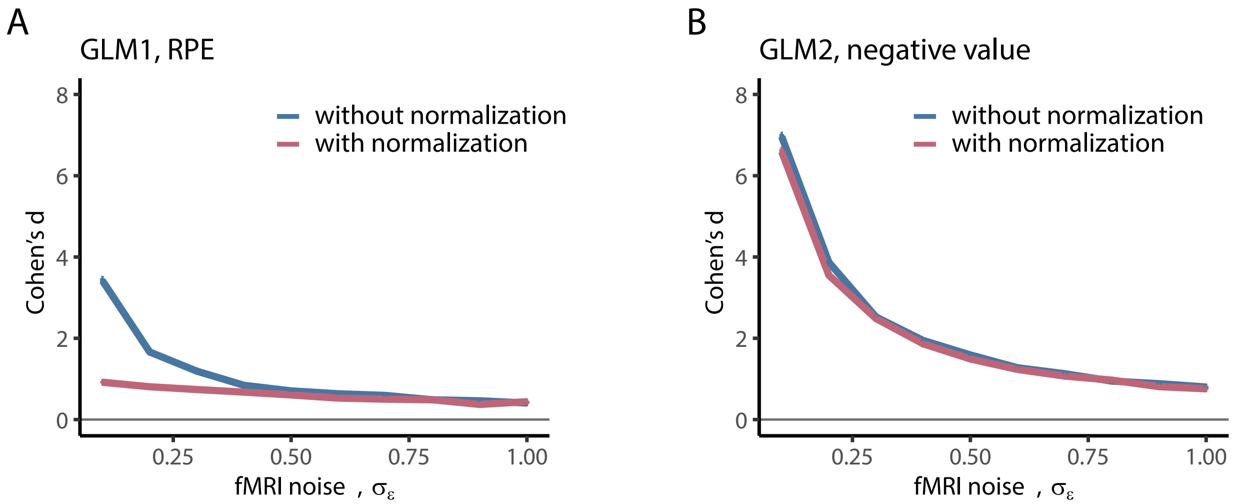

Fig 1. Effects of normalization of response variables and noise variance on between-group effect size of beta-values. The results without (blue) and with normalization (red) are plotted as a function of s.d. of fMRI noise. (A) Effect size for RPE of GLM1. (B) Effect size for negative value of GLM1.
