# Supplementary material for: Revisiting the importance of model fitting for model-based fMRI: It does matter in computational psychiatry
Source: PLoS Comput Biol. 2021 Feb 9;17(2):e1008738. doi: 10.1371/journal.pcbi.1008738 (PMC7899379; doi:10.1371/journal.pcbi.1008738)
Supplement: S6 Text — (PDF) [file pcbi.1008738.s006.pdf]

Supplementary Material (S6 Text)

Revisiting the importance of model fitting for model-based fMRI: It does matter in computational psychiatry

Kentaro Katahira[1], Asako Toyama[1]
[1]Department of Psychological and Cognitive Sciences, Nagoya University, Nagoya, Japan

Impacts of ignoring time courses of BOLD signals

Following Wilson & Niv [1], our simulations and analytical calculations treated latent variables themselves as regressors for GLMs used in model-based fMRI analysis (i.e., we performed simulation analysis in trial space rather than in continuous experimental time). In actual model-based fMRI, however, regressors for BOLD signals are usually constructed by convolving the impulse sequence with a hemodynamic response function (HRF), where the height of the stick function (impulse) is parametrically modulated by the variable of interest (e.g., RPE) and the timing of impulse is usually set at stimulus onset (e.g., when a reward is presented). Ignoring the time course of hemodynamic responses and the precise time course in each trial might be problematic because the convolution with HRF tends to increase correlations of signals from models at different parameter values. This could diminish the effects we are concerned with. For example, consider an extreme case where the height of impulses (latent variables) takes positive non-zero values, and trial-by-trial fluctuations are small. For such a case, even if the correlation of latent variables between different models is small, BOLD dynamics can have a large positive correlation between models with different parameters because BOLD signal decay to zero and become negative (i.e., overshoot).

Here we consider the impact of ignoring the precise time course of hemodynamic responses based on empirical data from Niv, Edlund, Dayan & O'Doherty [2]. The data contain BOLD signals from the nucleus accumbens (NAc)—a region in which RPE-related activity has been repeatedly reported—when subjects perform a reinforcement learning task. However, here we did not use the BOLD signal itself; we used only the behavioral data and the timing of stimulus onsets. We compared the situation where a common set of parameter estimates and the situation where a single parameter set were used to obtain trial-by-trial estimates of RPE signals for all subjects. The detailed description of the data and the procedure for analysis are provided in the final section of this text.

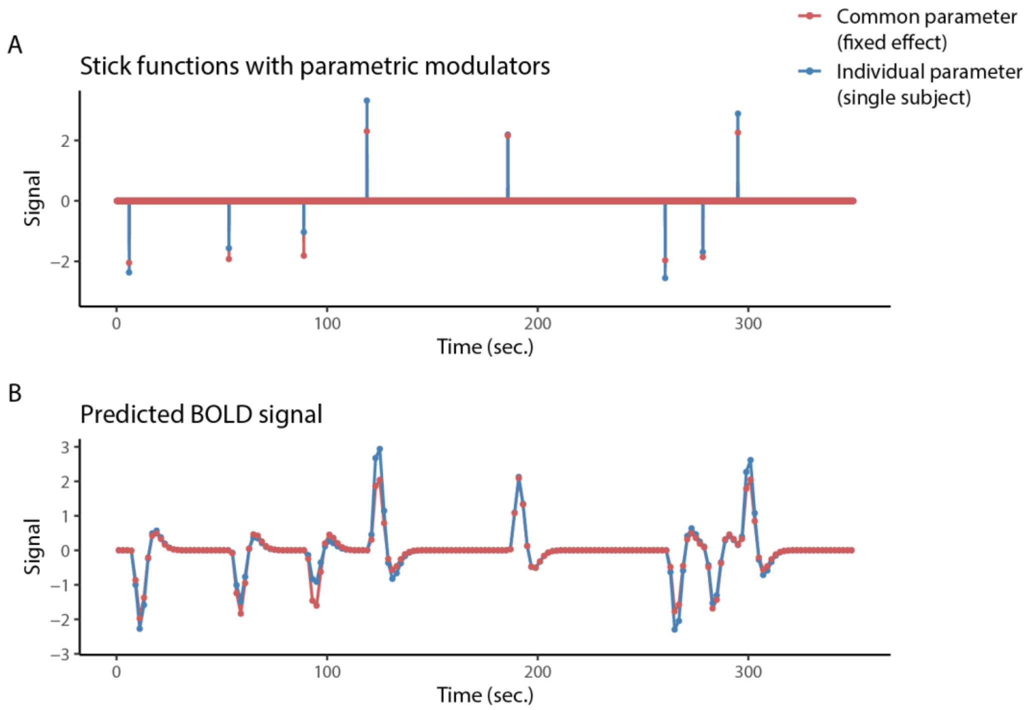

Fig 1. Example of the time course of regressors based on RPE signal from Niv et al. [2]. (A) Stick functions at decision outcome (reward) onsets. The heights of stick functions are parametrically modulated by RPEs derived from the reinforcement learning model whose parameters were obtained from fixed-effect estimation (red) and single-subject estimation (blue). (B) Regressors for BOLD signals, derived by convolving pulse sequences in panel A with HRF (two-gamma function). Note that RPE has a non-zero value only when the risky option (0/40) was chosen.

An example of impulse sequences with RPE being parametric modulators and resulting regressors with BOLD model are shown in Fig 1. These panels plot results obtained by using different estimation methods (fixed-effect estimation, which estimates a single parameter set to entire subjects, or single-subject estimation, which separately estimates for each subject).

The correlations between two estimation methods for raw latent variables and BOLD model are shown in Fig 2. The top panels show the result for RPE whose timings were set at outcome onset, and the bottom panels show the results for the chosen option value set at option stimulus onset. We observe that the correlation between different parameter estimation methods yielded a similar, but slightly large correlation for BOLD models (panels B and D).

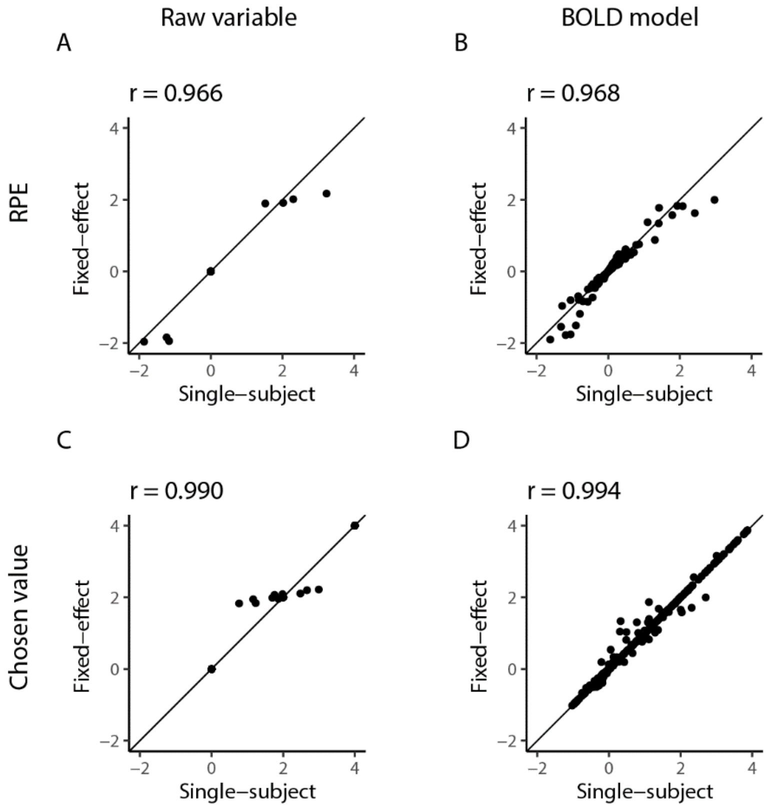

Fig 2. The correlations of regressors between models with different methods for parameter estimation (single-subject estimation vs. fixed-effect estimation) form a single session of a single subject. (A, C) Raw latent variable, in trial space. (B, D) BOLD signal model (convolved with the two-gamma function). The top two panels (A, B) show regressors of the RPE at outcome onset. The bottom two panels (C, D) show the value of the chosen option at choice option onset. Each dot represents a single time point.

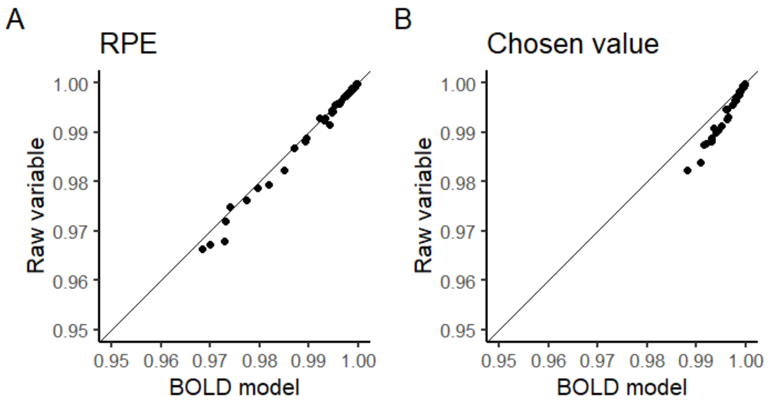

Fig 3. Comparison of correlation coefficients between estimation methods (raw variable vs. BOLD model). (A) RPE. (B) Value for the chosen option. Each dot represents the result of a single session of a single subject.

Fig 3 compares correlation coefficients between estimation methods for all three sessions of all 16 subjects. For RPE (panel A), the correlation coefficients are almost equivalent between raw variables and the BOLD model. For the value of the chosen option, the correlation is slightly higher for the BOLD model. Importantly, the relative relationship of correlation coefficients is largely unchanged across sessions and subjects. The session with a lower correlation in a raw latent variable leads to a smaller correlation also in the BOLD model. This indicates that regarding a raw latent variable as a regressor is sufficient for our purpose.

Material and methods for this supplementary text

We used data from Niv et al. [2]. The BOLD signal data after preprocessing and associated behavioral data were obtained from the original author's website [https://nivlab.princeton.edu/data]. Here, we briefly describe the experiment. For a more detailed description, we refer readers to the original paper [2].

Ethics statement

The experiment in Niv et al. [2] was approved by the Institutional Review Board of the California Institute of Technology. Participants gave informed consent in writing.

Behavioral task

In Niv et al. [2], 16 subjects performed a behavioral task in which five visual stimuli (drawings of slot machines) associated with a monetary reward were presented. There were two types of trials: choice trials in which subjects were required to choose between two stimuli and forced trials where subjects were presented with only one of the five stimuli and forced to choose it. After a stimulus was chosen, subjects could earn monetary outcomes depending on the chosen stimuli. Among five, two stimuli always yielded 0 cents (with probability 1.0), one always yielded 20 cents, another 40 cents, and one (the risky 0/40 stimulus) either 0 cents or 40 cents with a 0.5 probability for each.

Reinforcement learning model

Following Wilson & Niv [1], we used a standard reinforcement learning (RL) model

slightly simpler than the best-fitting model in Niv et al. [2], which has different learning rates for positive and negative RPEs. The value, V_t, is assigned to each option and referred to here as action values. After the presentation of decision-outcomes (reward or absence of reward), action values are updated according to the Rescorla-Wagner rule in Eq 1 and 2 in the main text. Based on the set of action values for presented stimuli in a choice trial, the model assigns the probability of choosing the option i using the soft-max function given in Eq 52 of the main text.

Fitting model parameters

To estimate the individual RL model parameters, we employed two parameter estimation methods: (1) single-subject (SS) estimation, where we fit each model parameter to each subject separately, and (2) fixed-effect (FE) estimation, in which a single parameter set was estimated for participants as a whole. In the single-subject estimation, we fit model parameters to each participant separately using maximum a posteriori (MAP) estimation. In MAP estimation, parameters that maximize the posterior probability density are used as estimates. To obtain the posterior probability density, we assigned the same prior distribution on each parameter, as in [2]. Specifically, a beta distribution with the parameters $a = 2$ and $b = 2$ was used as a prior for the learning rate, α, and a Gamma distribution with the shape parameter = 2 and scale parameter = 3 was used as a prior for the inverse temperature, β. For the fixed-effect estimation, maximum likelihood estimation (MLE), which searches a parameter set that maximizes the log-likelihood for all trials of all subjects, was performed. These estimations were performed using the rsolnp 1.16 package, which implements the augmented Lagrange multiplier method with an SQP interior algorithm. To facilitate finding the global optimum solution, algorithms were run 10 times; each run was initiated from a random initial value, and the parameter set that provided the lowest negative log likelihood was selected.

Constructing regressors for BOLD signal

The GLM for BOLD signal contains regressors composed of sets of stick functions, convolved with a standard two-gamma hemodynamic response function. Delta functions were parametrically-modulated, as described here. We analyzed the data separately with two independent GLMs for each RL parameter set (single-subject estimation, fixed-effect estimation). GLM was composed of temporal-difference (TD) errors as parametric modulators, consisting of two parametric modulators, the action value of the chosen option,

$V_t(i)$, was set at stimuli onset (slot machine), and the RPE, δ_t, set at outcome onset.

References

1. Wilson RC, Niv Y. Is Model Fitting Necessary for Model-Based fMRI? PLoS Computational Biology. 2015; 11(6):e1004237.

2. Niv Y, Edlund JA, Dayan P, O'Doherty JP. Neural Prediction Errors Reveal a Risk-Sensitive Reinforcement-Learning Process in the Human Brain. Journal of Neuroscience. 2012; 32(2):551–562.
