# Supplementary figures and images for: Revisiting the importance of model fitting for model-based fMRI: It does matter in computational psychiatry
Source: PLoS Comput Biol. 2021 Feb 9;17(2):e1008738. doi: 10.1371/journal.pcbi.1008738 (PMC7899379; doi:10.1371/journal.pcbi.1008738)

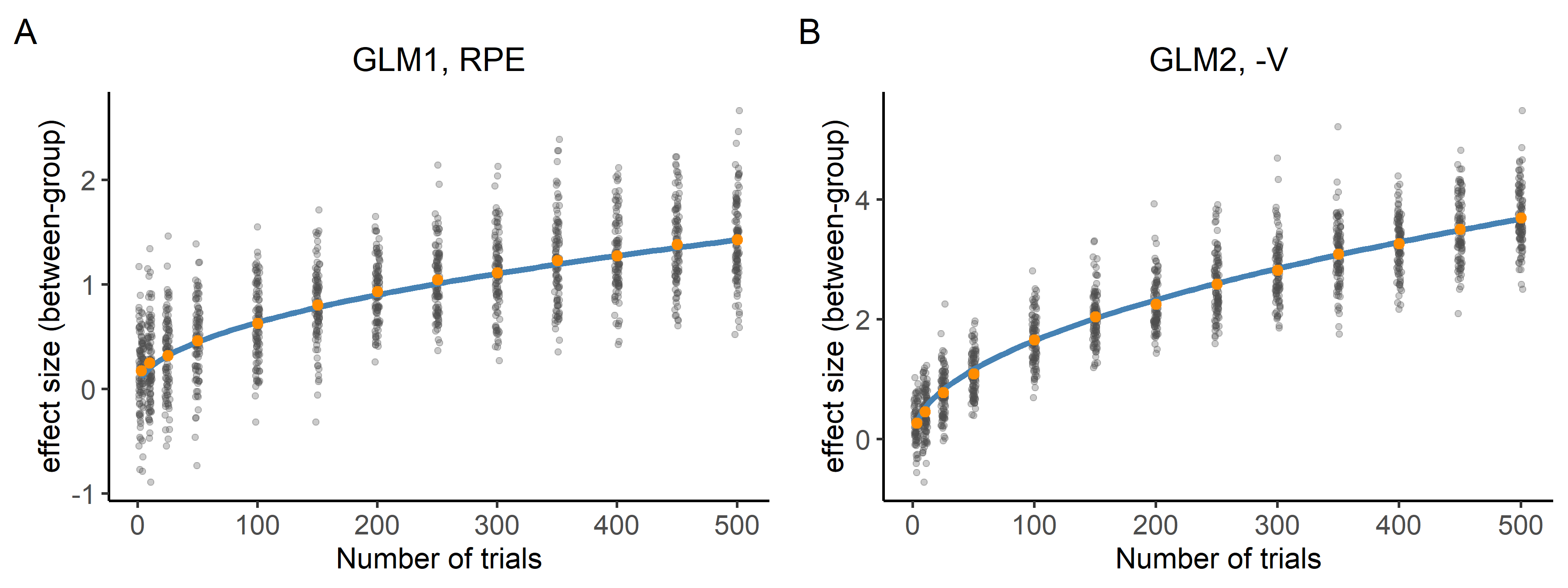

Supplement: S1 Fig — (A) For beta value of RPE in GLM1. (B) For beta value of negative value, −V, in GLM2. Gray dots represents the results of single simulation run. The number of trials was varied within 3, 10, 25, and from 50 to 500 with the step size 50. Except for that, the simulation setting is the same with those for Fig 2: We assumed the true learning rate of Low-L group (patient group) was α = 0.2, and that of High-L group (healthy control) was α = 0.4. The fit learning rate was α^=0.3. (PNG) [file pcbi.1008738.s007.png]

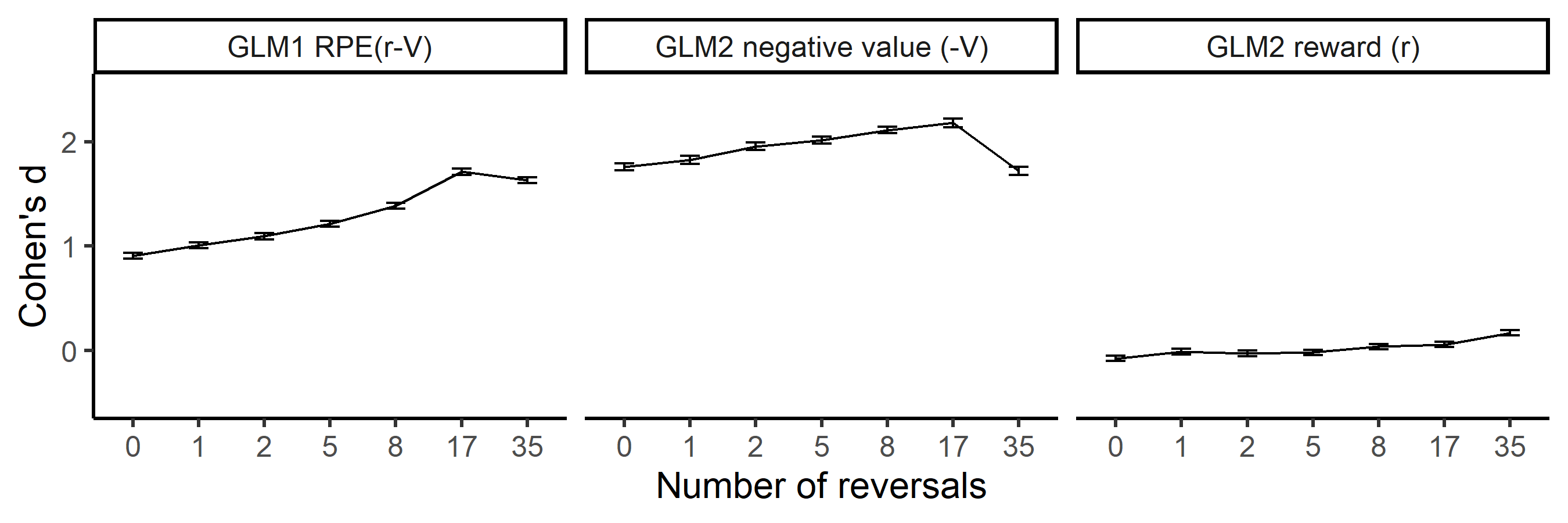

Supplement: S2 Fig — The effect size (Cohen’s d) for between-group difference of beta values are plotted as a function of the number of reversals of reward contingency. We simulated different frequencies of reward-contingency reversal (0, 1, 2, 5, 8, 17, or 35). For each reversal condition, we ran 100 simulations by producing anew reward history. Settings other than the reward contingency were the same with Fig 7 (“Effect of model-misspecification” section). This panel shows the mean effect size in each condition with error bars representing the standard error. For beta values of RPE in GLM1 and negative value in GLM2, the higher the frequency of the reversal, the larger the effect size. This reflects that the effect of the forgetting component has a larger effect on choice behavior when reversals are frequent. However, too many reversals (35 reversals in the panel) diminished effect size by ambiguating group differences in behavioral choices. (PNG) [file pcbi.1008738.s008.png]
